# Combination of DNA Prime – Adenovirus Boost Immunization with Entecavir Elicits Sustained Control of Chronic Hepatitis B in the Woodchuck Model

**DOI:** 10.1371/journal.ppat.1003391

**Published:** 2013-06-13

**Authors:** Anna D. Kosinska, Ejuan Zhang, Lena Johrden, Jia Liu, Pia L. Seiz, Xiaoyong Zhang, Zhiyong Ma, Thekla Kemper, Melanie Fiedler, Dieter Glebe, Oliver Wildner, Ulf Dittmer, Mengji Lu, Michael Roggendorf

**Affiliations:** 1 Institute of Virology, University Hospital of Essen, Essen, Germany; 2 Wuhan Institute of Virology, Chinese Academy of Sciences, Wuhan, People's Republic of China; 3 Department of Molecular and Medical Virology, Institute of Microbiology and Hygiene, Ruhr-University Bochum, Bochum, Germany; 4 Institute of Medical Virology, National Reference Centre for Hepatitis B and D Viruses, Justus-Liebig University, Giessen, Germany; 5 Hepatology Unit and Department of Infectious Diseases, Nanfang Hospital, Southern Medical University, Guangzhou, People's Republic of China; 6 Paul-Ehrlich-Institut, Division of Medical Biotechnology, Langen, Germany; Nationwide Children's Hospital, United States of America

## Abstract

A potent therapeutic T-cell vaccine may be an alternative treatment of chronic hepatitis B virus (HBV) infection. Previously, we developed a DNA prime-adenovirus (AdV) boost vaccination protocol that could elicit strong and specific CD8^+^ T-cell responses to woodchuck hepatitis virus (WHV) core antigen (WHcAg) in mice. In the present study, we first examined whether this new prime-boost immunization could induce WHcAg-specific T-cell responses and effectively control WHV replication in the WHV-transgenic mouse model. Secondly, we evaluated the therapeutic effect of this new vaccination strategy in chronically WHV-infected woodchucks in combination with a potent antiviral treatment. Immunization of WHV-transgenic mice by DNA prime-AdV boost regimen elicited potent and functional WHcAg-specific CD8^+^ T-cell response that consequently resulted in the reduction of the WHV load below the detection limit in more than 70% of animals. The combination therapy of entecavir (ETV) treatment and DNA prime-AdV boost immunization in chronic WHV carriers resulted in WHsAg- and WHcAg-specific CD4^+^ and CD8^+^ T-cell responses, which were not detectable in ETV-only treated controls. Woodchucks receiving the combination therapy showed a prolonged suppression of WHV replication and lower WHsAg levels compared to controls. Moreover, two of four immunized carriers remained WHV negative after the end of ETV treatment and developed anti-WHs antibodies. These results demonstrate that the combined antiviral and vaccination approach efficiently elicited sustained immunological control of chronic hepadnaviral infection in woodchucks and may be a new promising therapeutic strategy in patients.

## Author Summary

Chronic hepatitis B virus (HBV) infection is one of the major causes of liver cirrhosis and liver cancer worldwide. Recommended treatment regimens of chronic hepatitis B based on interferon alpha and nucleot(s)ide analogues do not lead to the satisfactory results. Over the last 20 years, continuous efforts have been undertaken to develop new immunotherapeutic approaches for the treatment of chronic hepatitis B, however, without satisfactory results. We proposed here that the combination of potent antivirals with a prime-boost vaccination protocol that is inducing appropriate virus-specific T-cell responses may restore immune control over HBV. To test this hypothesis we performed a proof-of-principle experiment using woodchucks, a widely accepted animal model of chronic HBV infection. We pretreated animals with entecavir to suppress viral replication and immunized them by a prime-boost regimen with DNA vaccines expressing woodchuck hepatitis virus (WHV) surface and core antigens and adenoviral vectors expressing WHV core antigen. Consistent with our hypothesis, the combination therapy achieved a stronger antiviral effect than the monotherapy alone, leading to sustained immunological control of chronic WHV infection and viral clearance in some animals. These data are encouraging and implicate the feasibility and usefulness of the immunotherapeutic strategies for the treatment of chronically HBV-infected patients.

## Introduction

Chronic hepatitis B virus (HBV) infection is still one of the major public health problems. Two billion people worldwide have been infected with HBV, of whom more than 360 million have developed chronic infection. Approximately one million patients die from HBV-associated liver diseases such as cirrhosis and hepatocellular carcinoma (HCC) every year.

Over the past 10 years, the treatment options of chronic HBV infection have improved greatly. Currently, the two types of antiviral therapies are approved: treatment with pegylated interferon alpha 2a (PEG-IFNα) or nucleot(s)ide analogues, such as entecavir (ETV) and tenofovir. However, these therapies have still several limitations. The treatment with PEG-IFNα leads to a sustained antiviral response in only one third of patients [1], regardless of combining the therapy with nucleot(s)ide analogues [2], and it is frequently associated with serious side effects. The treatment with nucleot(s)ide analogues significantly suppresses HBV replication but cannot completely eradicate the virus. After withdrawal of the treatment, a rebound of viremia is observed in the majority of patients. Therefore, the alternative strategies to treat chronic HBV infection are still urgently needed.

The host immune response determines whether acute HBV infection will progress to resolution or chronicity. An early and multi-specific immune response to HBV antigens is associated with the clearance of HBV [3], [4]. In contrast, a weak or often undetectable HBV-specific immune response correlates with HBV persistence [5]–[8]. Thus, it is assumed that therapeutic vaccination could enhance the virus-specific immune responses contributing to control or even clearance of chronic HBV infection. Early therapeutic vaccines were based on the recombinant HBV surface antigen (HBsAg) protein vaccines [9]–[19]. These vaccines proved to be excellent in their prophylactic potential, but were unfortunately not effective in chronically infected patients. A DNA vaccine expressing small and middle HBV envelope proteins was also tested in chronic HBV carriers but failed to elicit sustained HBV-specific cellular immune response [20]. Moreover, administration of this vaccine to HBV carriers pre-treated with nucleoside analogues did not induce any therapeutic effect in a recent clinical trial (*S. Pol, personal communication*).

A vigorous T-cell response against HBV core antigen is crucial for the resolution of the infection but is predominantly absent in chronic hepadnaviral infections [21]–[26]. Thus, using T-cell vaccines targeting the core protein may be a potent therapeutic strategy. We hypothesized that improved WHcAg-based T-cell vaccines might be crucial to achieve sustained antiviral immunological responses. Therefore, we developed new vaccines in the woodchuck model, a proven preclinical model to study innovative prophylactic and therapeutic strategies against HBV infection. We constructed a new DNA plasmid (pCGWHc) and adenoviral vectors serotype 5 (Ad5WHc) and chimeric Ad5 displaying Ad35 fiber (Ad35WHc) showing high expression levels of WHcAg. These new vaccines were tested recently in mice and naïve woodchucks [27]. We showed that the DNA prime – AdV boost immunization significantly improved the magnitude of WHcAg-specific T-cell responses in mice, far beyond the previously tested by us strategies. Moreover, for the first time, we were able to induce detectable WHcAg-specific proliferative and cytotoxic T-cell responses in naïve woodchucks using these optimized vaccines. We demonstrated that heterologous Ad5WHc-Ad35WHc regimen was superior in priming of WHcAg-specific T-cell responses, compared to only DNA immunization in the woodchuck model [27]. Nevertheless, using of only recombinant adenoviruses in vaccinations regime limits the number of immunizations, due to induction of neutralizing antibodies against the structural components of the vector. Therefore, DNA prime-AdV boost immunization seems to be a rational approach which combines increased efficacy of the vaccination regime with the possibility of using multiple immunizations necessary to break immune tolerance to the targeted antigens in chronically-infected individuals.

In the present study we evaluated the optimized DNA prime – AdV boost immunization first in WHV transgenic mice and then in chronically WHV-infected woodchucks. The WHV transgenic mouse, carrying a 1.3 fold overlength WHV transgenome, is a new animal model with well established immunological tools to determine virus-specific T-cell response. WHV replication occurs specifically in the liver and WHV particles could be produced and released into the bloodstream. We found that WHV transgenic mice are not completely tolerant to WHV proteins and WHV-specific T-cell responses could be primed by DNA vaccines, though at a low level (*our unpublished results*). Thus, this model was ideal to evaluate our new prime-boost immunization regimen prior to the therapeutic vaccination experiment performed in chronically WHV-infected woodchucks. We could show that our new immunization strategy was able to induce effective immune responses to WHV antigens and reduce WHV replication WHV transgenic mice. In chronically WHV-infected woodchucks we combined WHcAg-based DNA prime-AdV boost vaccinations with WHsAg-expressing plasmid, in order to achieve the most favorable therapeutic effect. Following the idea that the reduction of viral loads by the nucleoside analogues pre-treatment could enhance the effect of therapeutic immunization [28]–[30], we used a potent antiviral drug entecavir (ETV). In contrast to lamivudine therapy [31], [32], treatment with ETV proved to efficiently suppress WHV replication in chronically infected woodchucks [33], however, does not lead to the resolution of the infection. The results showed that our new combination therapy improved WHV-specific immune responses and led to long term viral control, induction of neutralizing anti-WHs antibodies, and viral clearance in some animals.

## Results

### The DNA prime – AdV boost breaks the immune tolerance against WHV antigens in WHV transgenic mice

We have shown recently that immunization of naïve C57BL/6 mice in heterologous DNA prime – AdV boost manner using vaccines expressing WHcAg induces remarkably vigorous and potent WHcAg-specific response. [27]. We investigated whether this new vaccination protocol is able to break the WHV-specific immune tolerance and reduce the WHV replication in WHV transgenic mouse model. Thereby, mice were primed twice with the pCGWHc plasmid in a two-week interval and afterwards were boosted once with Ad5WHc or pCGWHc, or boosted twice with Ad5WHc followed by Ad35WHc.

**Figure 1:**
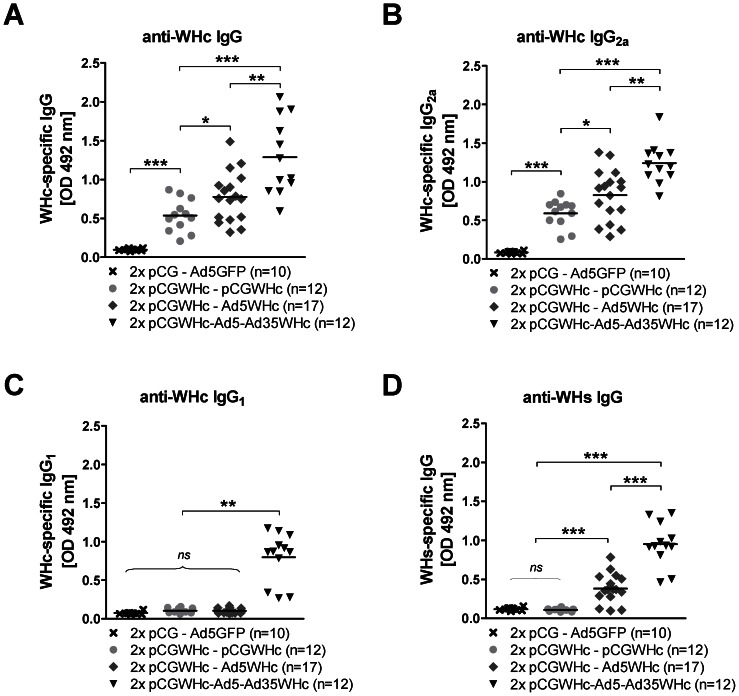
Detection of WHV-specific antibodies induced by DNA prime – AdV boost immunization in WHV transgenic mice. Mice were primed two times by immunization with the pCGWHc plasmid. Four weeks later boosting immunization with Ad5WHc, Ad35WHc, or pCGWHc was performed. The group of mice immunized twice with pCGWHc in combination with Ad5WHc was boosted 4 weeks later for a second time with Ad35WHc. Mice immunized with ‘empty’ pCG and boosted with Ad5 expressing GFP served as controls. WHcAg-specific IgG (A), IgG_2a_ (B), IgG_1_ (C) or WHsAg-specific IgG (D) antibodies were detected in sera collected two weeks after the last immunization (serum dilution 1∶500). Asterisks mark the significant difference (*<0.05; **<0.005, ***<0.0005; *ns* – not significant).

As shown in Fig. 1A, the levels of WHcAg-specific antibodies (anti-WHc) were significantly higher in WHV Tg mice that received boosting immunization with Ad5WHc than in group of mice immunized three times with DNA vaccine (*P*<0.05). The level of anti-WHc increased additionally in the group of mice after the fourth (second boost) immunization with Ad35WHc (*P*<0.005). As expected control mice mice did not induce any anti-WHc antibodies (*P*<0.0005, compared to all groups of immunized mice). The levels of anti-WHc antibodies were comparable in all mice immunized with pCGWHc plasmid either once or twice (*data not shown*). Detection of IgG isotypes demonstrated that all tested immunization protocols induced predominantly IgG_2a_ antibodies (Fig. 1B). The anti-WHc antibodies of IgG_1_ subclass were only observed in group of mice immunized in DNA-Ad5WHc-Ad35WHc manner (Fig. 1C) (*P*<0.005, compared to other vaccination groups). Interestingly, we could detect WHsAg-specific antibodies (anti-WHs) in groups of mice that were immunized with DNA prime – AdV boost regimens, but not in mice immunized only with DNA (Fig. 1D). The anti-WHs antibodies were detected in the sera of 14 out of 17 mice after the boosting immunization with Ad5WHc (*P*<0.005). The levels of anti-WHs increased additionally after the second boosting immunization with Ad35WHc (*P*<0.005). The control experiment in WHV transgenic mouse strain 1218 (harbouring a mutated WHV transgenome lacking WHsAg) showed that DNA-Ad5WHc-Ad35WHc immunization induces the same pattern of WHc-specific antibodies. However, no anti-WHs were detected after Ad35WHc immunization (supplementary Fig.S1). These data indicate that a potent immunization with WHcAg is able to boost a weak anti-WHs response by intermolecular help mechanism if WHsAg is present [34].

**Figure 2:**
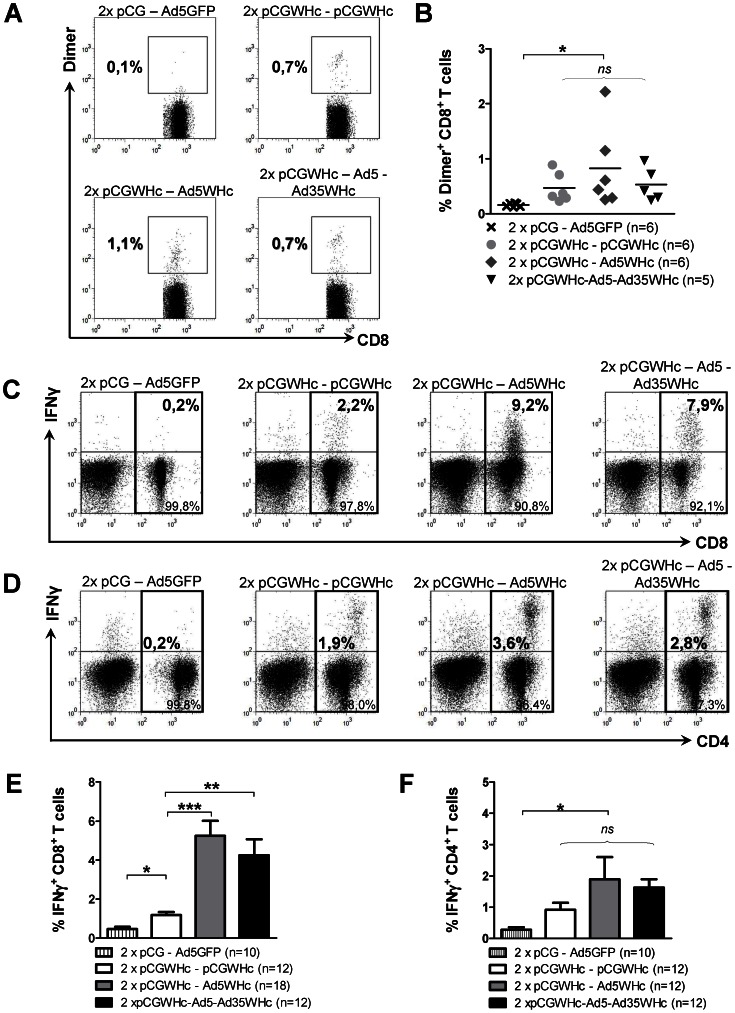
Cellular immune response induced by DNA prime – AdV boost immunization in WHV transgenic mice. Mice were primed two times by immunization with the pCGWHc plasmid. Four weeks later boosting immunization with Ad5WHc, Ad35WHc, or pCGWHc was performed. The group of mice immunized twice with pCGWHc in combination with Ad5WHc was boosted 4 weeks later for a second time with Ad35WHc. Mice immunized with ‘empty’ pCG and boosted with Ad5 expressing GFP served as controls. (A–B) Representative and summarised frequencies of WHcAg-specific CD8^+^ T-cells detected *ex vivo* in the population of splenic lymphocytes. Antigen-specific cells were detected using DimerX H2-Db fusion protein loaded with H2-Db-restricted CD8^+^ epitope c13-21. (C,E) Representative and summarised WHcAg-specific IFNγ^+^ CD8^+^ T-cell responses detected in the splenocytes expanded *in vitro* for 7 days with CD8^+^ T-cell epitope c13-21. (D,F) Representative and summarised WHcAg-specific IFNγ^+^ CD4^+^ T-cell responses in the splenocytes expanded *in vitro* for 7 days with CD4^+^ T-cell epitope c131-145. The bars represent the mean value obtained for each group of mice including SEM. Asterisks mark the statistically significant difference (*<0.05;**<0.005; ***<0.0005; *ns* – not significant).

In the next step, we evaluated the impact of DNA only, heterologous DNA–Ad5WHc, or DNA–Ad5WHc-Ad35WHc regimens on induction of cellular immune response 2 weeks after the last immunization. In the first step, we assessed the presence of antigen-specific T-cells within the splenic lymphocytes *ex vivo*, using WHcAg-derived peptide c13-21-specific dimer. As Fig. 2A shows, we could detect WHcAg-specific CD8^+^ T-cells in the spleens of mice immunized with WHcAg-expressing vaccines but not in ‘empty’ pCG-Ad5GFP control group. The mean background values of the assay obtained for these controls was 0.16% and was comparable to 0.15% detected in the isotype controls (*data not shown*). The mean values of WHcAg-specific CD8^+^ T-cells in the spleens of mice immunized with DNA-only was 0.47%, with DNA-Ad5WHc was 0.83%, and with DNA-Ad5WHc-Ad35WHc was 0.52% (Fig. 2B) (*P*<0.05, compared to controls).

The magnitude of the WHcAg-specific CD8^+^ and CD4^+^ T-cell responses elicited by the various vaccination regimens was compared by the intracellular IFNγ staining of splenocytes. Splenocytes were isolated two weeks after the last immunization and were stimulated *in vitro* for 7 days with the CD8^+^ T-cell epitope c13-21. The percentages of IFNγ^+^ CD8^+^ T-cells determined in the spleens of mice vaccinated in the DNA prime – Ad5WHc boost manner (mean 5.25%) were significantly higher in comparison to the only DNA-immunized group (mean 1.18%; *P*<0.0005) (Fig. 2C,E). Unexpectedly, the magnitude of IFNγ response did not increase in the group of mice that received the second boosting immunization with Ad35WHc. The mean percentages of IFNγ^+^ CD8^+^ T-cells directed against c13-21 in this group were 4.26% and were slightly lower than in the DNA-Ad5WHc group. All immunization protocols were able to induce significant IFNγ secretion by CD4+ T-cells in response to stimulation with peptide c131-145 (*P*<0.05) (Fig. 2D). Nevertheless, no statistically significant difference in percentages of IFNγ^+^ CD4^+^ T-cells between the groups immunized with WHcAg-expressing vaccines was detected (Fig. 2F).

**Figure 3:**
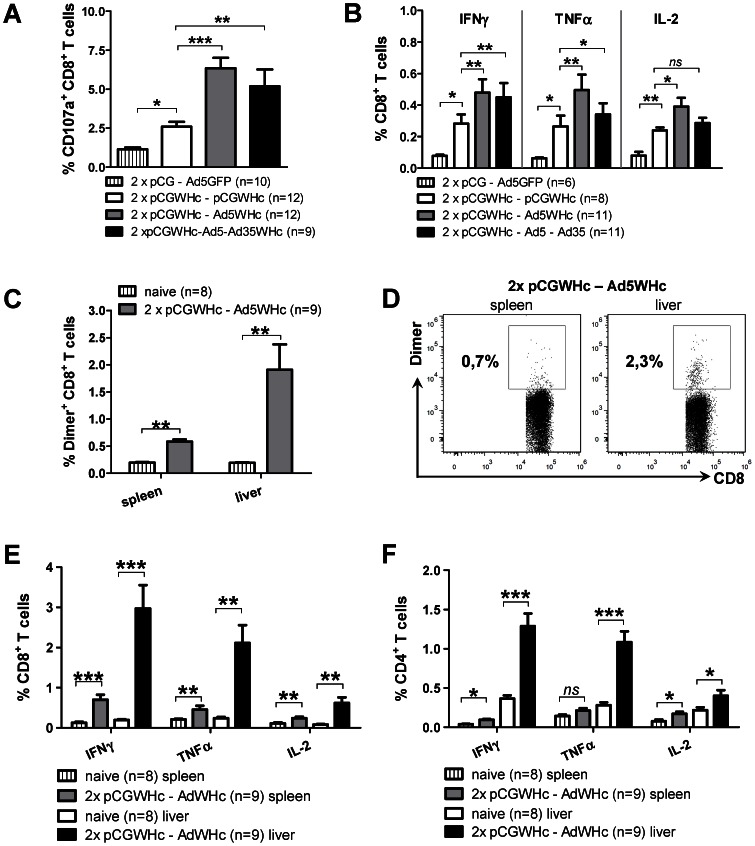
Functional analysis of splenic and hepatic WHV-specific T-cells elicited by vaccinations in WHV transgenic mice. (A) Degranulation capacity of IFNγ^+^ CD8^+^ splenic T-cells expanded *in vitro* for 7 days with peptide c13-21. (B) Frequencies of IFNγ- TNFα- and IL-2-producing CD8^+^ T-cells detected *ex vivo* in splenocytes stimulated for 6 h with the epitope c13-21. (C) Summary of WHcAg-specific CD8^+^ T-cells detected *ex vivo* in the populations of splenic and hepatic lymphocytes. (D) Representative dot-plots of WHcAg-specific CD8^+^ T-cells detected *ex vivo* in the spleen and the liver of one DNA prime – AdV boost immunized WHV transgenic mouse. (E–F) Frequencies of IFNγ- TNFα- and IL-2-positive CD8^+^ and CD4+ T cells detected ex vivo in splenic and hepatic lymphocytes stimulated 6 h with CD8^+^ T-cell epitope c13-21 or CD4^+^ T-cell epitope c131-145, respectively. The bars represent the mean value obtained for each group of mice including SEM. Asterisks mark the statistically significant difference (*<0.05;**<0.005; ***<0.0005; *ns* – not significant).

Further on, we compared the effector functions of the CD8^+^ T-cells induced by the various immunization regimens, such as degranulation capacity and secretion of the other antiviral cytokines e.g. TNFα and IL-2. The ability of the CD8^+^ T-cells to degranulate was measured by flow cytometric detection of the CD107a marker [35], [36] on the surface of the lymphocytes expanded *in vitro* for 7 days with the c13-21 epitope. The results show that the percentages of CD107a^+^ CD8^+^ T-cells were significantly higher (*P*<0.005) in the groups of mice boosted once with Ad5WHc or twice with Ad5WHc and Ad35WHc (mean values: 6.3% and 5.2%, respectively), than in the pCGWHc only immunized mice (mean value: 2.6%) (Fig. 3A).

The production of T_H_1 type cytokines by CD8^+^ T-cells, such as IFNγ, TNFα and IL-2, was evaluated in the splenocytes *ex vivo*, after 6 h stimulation with the peptide c13-21. As presented in Fig. 3B, all vaccination protocols induced significant percentages of CD8^+^ T-cells positive for all of the tested cytokines compared to mean background values (0.06%–0.08%) detected in mice immunized with ‘empty’ pCG plasmid and boosted with Ad5GFP (*P*<0.05). The percentages of IFNγ^+^ or TNFα^+^ CD8^+^ T-cells were detectable at similar levels and the percentages of IL-2^+^ CD8^+^ T-cells were at slightly lower levels. Group of mice primed with pGCWHc and once boosted with Ad5WHc showed the highest percentages of IFNγ^+^, TNFα^+^, and IL-2^+^ CD8^+^ T-cells (the mean values: 0.48%, 0.49%, and 0.39% respectively). The frequencies of CD8^+^ T-cells positive for all of the tested cytokines in this group (2×pCGWHc-Ad5WHc) were significantly higher compared to the DNA only - immunized group (the mean values: 0.28%, 0.26% and 0.24%, respectively) (*P*<0.05). Mice immunized four times with DNA-Ad5WHc-Ad35WHc regimen exhibited significantly higher frequencies of only IFNγ-positive and TNFα-positive CD8^+^ T-cells compared to the DNA only – immunized group (the mean values: 0.45% and 0.34%, respectively).

In addition, we analysed the effector functions of hepatic WHcAg-specific T-cells induced by 2×pCGWHc-Ad5WHc immunization, as the liver is the major compartment of WHV replication in WHV transgenic mice. First, we evaluated the presence of hepatic antigen-specific T-cells *ex vivo*, using WHcAg-derived peptide c13-21-specific dimer. As shown in Fig. 3C, we detected WHcAg-specific CD8+ T-cells in the liver of mice immunized with the DNA prime – Ad5WHc boost regimen, but not in naïve WHV transgenic mice (*P*<0.005). The mean frequencies of WHcAg-specific CD8^+^ T-cells in the liver of immunized mice was 1,9%. Moreover, the percentages of dimer^+^ CD8^+^ T-cells in the liver were significantly higher compared to these detected in the spleen (0,6%; *P*<0.05) (Fig. 3C–D).

The frequencies of CD8^+^ and CD4^+^ T-cells producing IFNγ, TNFα and IL-2 (detected *ex vivo* after 6 h stimulation with the CD8^+^ or CD4^+^ T-cell epitopes), were also higher in the liver than in the spleen of mice immunized with 2×DNA-Ad5WHc regimen. As presented in Fig. 3E, the immunization induced significant percentages of IFNγ^+^, TNFα^+^ or IL-2^+^ CD8+ T-cells (2,97%, 2,11%, 0,62%, respectively) compared to mean background values detected in naïve WHV transgenic mice (0,20%, 0,24%, 0,08%, respectively) (*P*<0.005). Similarly, the mean frequencies of IFNγ^+^, TNFα^+^ or IL-2^+^ CD4^+^ T-cells in immunized mice (1,30%, 1,10%, 0,40%, respectively) were significantly higher, compared to the corresponding values detected in the naïve mice (0.37%, 0.28% and 0.20%, respectively) (*P*<0.05) (Fig. 3F).

### The immunization of 1217 WHV Tg mice by DNA-AdV prime-boost regimen leads to a significant reduction in the viral loads

**Figure 4:**
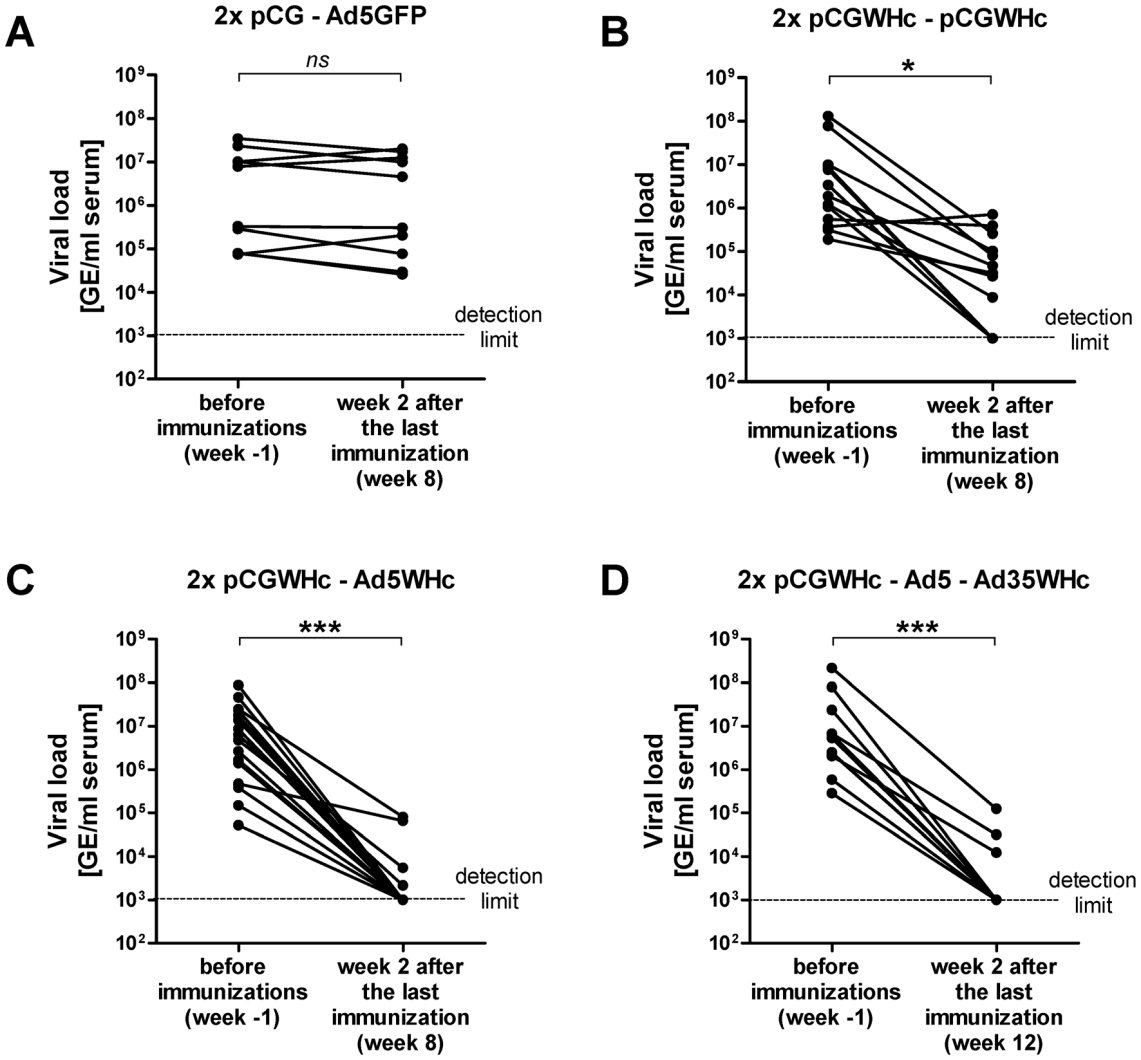
Quantification of the WHV loads before and after the immunization trials in WHV transgenic mice. The viral load was evaluated by quantitative real-time PCR with the detection limit of 10^3^ genome equivalents (GE) per milliliter serum. For analysis DNA samples obtained from the serum of mice were used. The single pair of dots connected with the line represents values obtained from one mouse before (time point of cardiotoxin pretreatment) and after the immunization trials (week 2 after the last immunization). The correlations of the WHV loads for 4 immunization groups of mice are presented: (A) control group primed with ‘empty’ pCG and boosted with Ad5 expressing GFP, (B) group immunized only with DNA vaccine – pCGWHc, (C) group primed with pCGWHc and boosted with Ad5WHc, and (D) group primed with pCGWHc and boosted twice with Ad5WHc and Ad35WHc. The statistical analysis was performed using the Wilcoxon signed rank test (*<0.05; ***<0.0005; *ns* – not significant).

We examined the impact of the WHcAg-based immunizations on the WHV replication in 1217 WHV Tg mice. The viral loads were monitored in the serum of mice before the immunizations were performed (week −1) and afterwards, at the time point of sacrifice (week 8 for single boost groups and 12 for double boost, respectively). As expected, in the control group of mice no difference in the viral loads in serum at the beginning and at the end of the experiment was observed (Fig. 4A). In the group immunized three times with plasmid DNA – pCGWHc only, four out of twelve mice (33%) had undetectable viral loads at the end of the experiment (Fig. 4B). Other mice except two, showed significant 1 to 2 log decrease in viral load after the immunizations (*P*<0.05). As showed in Fig. 3C–D, mice immunized in the heterologous prime – boost manner using recombinant adenoviral vectors demonstrated the most significant reduction in viral loads (*P*<0.0005). At the end time point, the WHV DNA was undetectable in 13 out of 17 mice from the group boosted once with Ad5WHc (77%) (Fig. 4C). In the group of mice that received the fourth immunization with Ad35WHc, nine out of twelve mice (75%) exhibited the WHV viremia below the detection limit at the end of the experiment (Fig. 4D).

### Heterologous prime-boost immunization in combination with ETV leads to induction of significant WHV-specific T-cell response in treated chronic WHV carriers

**Figure 5:**
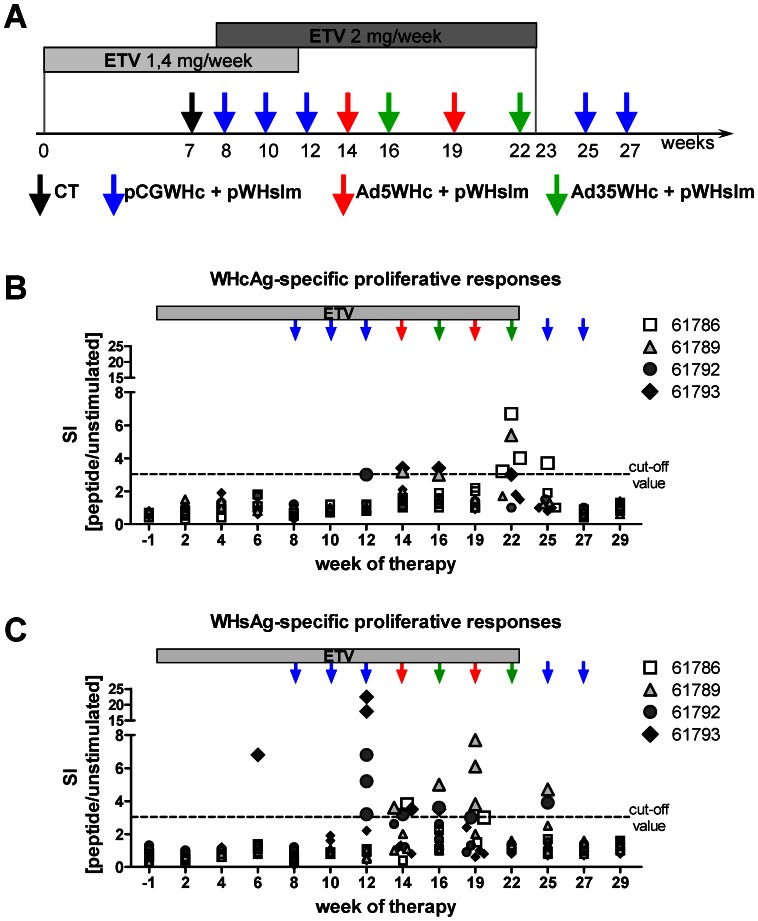
WHV-specific lymphoproliferative responses detected in chronically WHV-infected woodchucks treated with the combination therapy. (A) Immunization schedule. Six chronically WHV-infected woodchucks (number: 61786, 61789, 61791, 61792, 61793 and 61795) were treated with entecavir for 23 weeks. The drug was administered for 12 weeks in a dose of 1.4 mg ETV per week. From week 8 to 23 of the therapy, subcutaneous injections of 1 mg ETV were performed twice a week. At week 7, four of the six ETV-treated animals (number: 61786, 61789, 61792, and 61793) were pretreated with cardiotoxin (black arrow) and one week later the animals received in total 9 intramuscular subsequent immunizations with 0,5 mg of pCGWHc together with 0,5 mg of pWHsIm (time points of immunization marked by the blue arrows at weeks: 8, 10, 12, 25 and 27), 1×10^10^ PFU of Ad5WHc together with 0,5 mg of pWHsIm (red arrows at weeks 14 and 19) or 1×10^10^ PFU of Ad35WHc together with 0,5 mg of pWHsIm (green arrows at weeks 16 and 22). Two animals (number 61791 and 61795) were treated only with ETV and served as controls. WHcAg-specific (B) and WHsAg-specific (C) lymphoproliferative responses in vaccinated woodchucks. The PBMCs were stimulated with panel of 10 WHcAg-specific and 16 WHsAg-specific peptides in triplicates. After 5 days of culture, cells were pulsed with ^2^[^3^H]adenine for 16 h and the incorporation of ^2^[^3^H]adenine was measured. Results for triplicate cultures are presented as a mean stimulation index (SI). A SI≥3.0 was considered significant.

We evaluated the effectiveness of heterologous DNA prime – AdV boost immunization as the therapeutic vaccine in chronically WHV-infected woodchucks. To increase the effect of the vaccination we used antiviral pretreatment with entecavir to reduce the WHV replication. The drug was administered for 23 weeks. Starting from week 8, four animals received in total 9 sequential intramuscular immunizations with DNA plasmids expressing WHcAg and WHsAg, Ad5WHc, and Ad35WHc as shown in Fig. 5A. Two animals treated only with ETV served as controls. We included WHsAg expressing plasmid into the vaccination schedule, as the results obtained in WHV transgenic mice demonstrated that a certain amount of WHsAg is necessary to stimulate B-cells to produce anti-WHs antibodies (Fig. 1; Fig.S1). Therefore, including WHsAg as a part of vaccine may increase the immunotherapeutic effect. Moreover, after the administration of adenoviral vectors, we performed two additional DNA immunizations to maintain the induced WHV-specific T-cell responses.

The WHV-specific T helper (T_H_) response was evaluated by 2[^3^H]adenine-based proliferation assay of woodchuck PBMCs stimulated with the known T_H_ epitopes The significant WHV-specific proliferative responses (SI≥3.0) were detectable in PBMCs of all chronically WHV-infected woodchucks that received the vaccinations (DNA/Ad5WHc/Ad35WHc) and ETV (Fig. 5B–C), but not in animals treated only with ETV (SI values ranging from 0.3 to 2.3 for all examined peptides, at all tested time points; *data not shown*). Except of woodchuck 61793, which showed a transient response against WHsAg-derived peptide 336–351 (SI = 6.8) shortly after beginning of ETV treatment (week 6), the other woodchucks from the combination therapy demonstrated the proliferation of virus-specific T-cells during the immunizations. Two out of four vaccinated woodchucks (number 61792 and 61793) showed significant proliferative responses already after two immunizations with plasmid DNA vaccine (week 12 of therapy). The detected responses were directed against WHsAg-derived peptides: s224-239, s252-263, or s420-431 (SI ranging from 3.2 to 22.4) (Fig. 5C) and one WHcAg-derived peptide c85-100 (SI = 3.0; woodchuck 61792) (Fig. 5B) After three DNA injections (week 14 of therapy), all four immunized woodchucks showed significant proliferative responses against WHsAg-derived peptides (SI ranging from 3.2 to 3.8). In addition, 2 out of 4 woodchucks demonstrated WHcAg-specific T_H_ responses. The WHV-specific proliferative responses were present in most of the woodchucks until week 25 (two weeks after the last ETV treatment and 3 weeks after the second Ad35WHc/pWHsIm immunization). We identified 5 WHsAg-specific and 5 WHcAg-specific T-cell epitopes (see supplementary Table S1). The WHsAg-specific proliferation was predominantly directed against peptides s224-239 and s252-267. The most frequently recognized WHcAg-derived peptides were: c64-79 and c117-132.

**Figure 6:**
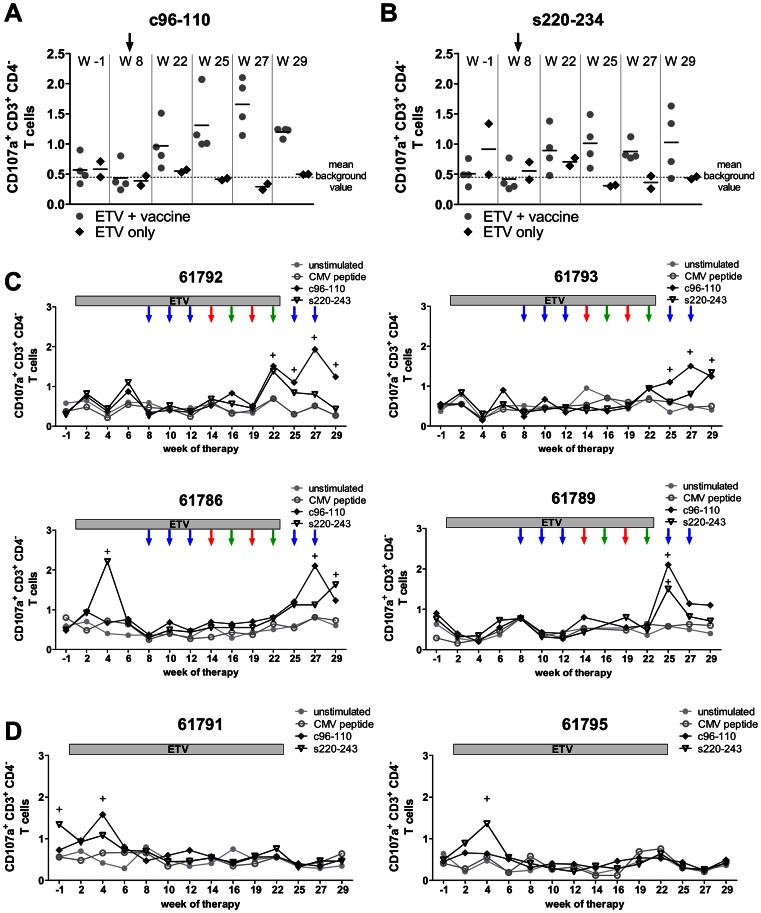
Cytotoxic T-cell responses detected in chronically WHV-infected woodchucks treated with the combination therapy. Summary of WHcAg-specific (A) and WHsAg-specific (B) degranulation responses evaluated by CD107a assay at representative time points of the experiment. PBMCs were expanded *in vitro* for 3 days with WHcAg-derived epitope c96-110 or WHsAg-derived epitope s220-234. The background value was calculated as a mean of all values detected for negative controls in all woodchucks at all time points. Presented values show the percentage of CD107a^+^ CD3^+^ CD4^−^ T-cells in the CD3^+^ CD4^−^ T-cell population. The black arrow represents beginning of the immunization regimen at week 8. (C–D) The kinetics of CD107a^+^ degranulation responses in WHV chronic carriers at all monitored time points of therapy. Chronically WHV-infected woodchucks were treated with ETV for 23 weeks. Four of them received subsequently 9 intramuscular immunizations with DNA plasmids, expressing WHcAg and WHsAg (blue arrows), Ad5WHc (red arrows), and Ad35WHc (green arrows). Two animals (number 61791 and 61795) were treated only with ETV and served as controls. The positive CTL responses are marked with “+” sign.

The evaluation of cytotoxic T-cell response against previously identified epitopes c96-110 and s220-234 [37] was performed by CD107a degranulation assay. The population of CD3^+^ CD4^−^ lymphocytes was considered to be the CD8^+^ T-cells as there is no specific anti-woodchuck CD8^+^ antibody. The WHV-specific degranulation responses were not detectable in most of the animals before week 22 of the treatment. At week 4 of ETV therapy, only a brief elevation in percentages of WHcAg- and WHsAg-specific CTLs were observed in three WHV chronic carriers (woodchucks 61786, 61791, and 61795), as shown in Fig. 6. This result indicates that a decrease of WHV replication by entecavir treatment is accompanied by a transient restoration of T-cell functions. All woodchucks from the combination therapy group and control animals had comparable background percentages of WHcAg- and WHsAg-specific CTLs at the beginning of the immunization phase (week 8) (Fig. 6A–B). The WHcAg-specific T-cell responses appeared in two immunized woodchucks 61792 and 61793 at week 22 (Fig. 6A,C). The percentages of 1.51% and 1.43% of WHcAg-specific CTLs detected for woodchuck 61792 and 61793, were three fold higher than the mean background value of 0.43% calculated for the negative controls (unstimulated cells and cells stimulated with unrelated CMV-derived peptide) of all woodchucks at all time points. The WHcAg-specific degranulation response was present in all 4 woodchucks from combination therapy group until the last monitored time point week 29. The peak of WHcAg-specific CTLs detected in peripheral blood of the immunized woodchucks was detected at week 27 of treatment; the percentages of CD107a^+^ CD3^+^ CD4^−^ T-cells were ranging between 1.2%–2.1% (mean: 1.7%). The ETV only treated controls did not show any significant WHcAg-specific T-cell response (Fig. 6D). The mean percentages of WHcAg-specific CTLs detected from week 22 to 29 were ranging between 0.29% and 0.55% and were comparable with the mean background value (0.43%). The WHsAg-specific CTL responses were not as prominent as the responses directed against WHcAg and appeared only transiently (Fig. 6C). Nevertheless, woodchucks that received the combination therapy demonstrated higher percentages of WHsAg-specific CTLs (mean 0.9–1.0%, weeks 25–29) in comparison to background values detected for only ETV treated animals (mean 0.3–0.4, weeks 25–29) (Fig. 6B). The peak of WHsAg-specific degranulation response detected in peripheral blood of WHV chronic carriers that received immunization differed in time. At week 22 the highest percentages of WHsAg-specific CTLs were detected for 61792 (1.38%), at week 25 for 61789 (1.49%), at week 29 for 61786 and 61793 (1.63 and 1.34% respectively). The representative dot-plots of WHcAg- and WHsAg-specific CD107a degranulation responses are shown in supplementary Fig. S2.

### Effect of heterologous prime-boost immunization in combination with ETV on viral replication and markers of chronic infection

We investigated the impact of the combination therapy on WHV replication, WHsAg levels, development of anti-WHs antibodies, and liver inflammation by measurements of liver transaminase - GOT. The baseline values of the viral loads prior to ETV therapy in WHV chronic carriers enrolled in the experiment was ranging from 3.1×10^9^ to 1.2×10^11^ WHV GE/ml of serum. The initial levels of WHsAg significantly varied between the individual animals and were ranging from 96.20 µg/ml (woodchuck 61792) to 693.83 µg/ml serum (woodchuck 61786). Only two woodchucks 61786 and 61795 demonstrated elevated GOT level in the serum at the beginning of the experiment ETV treatment (67, and 150 IU/l, respectively).

**Figure 7:**
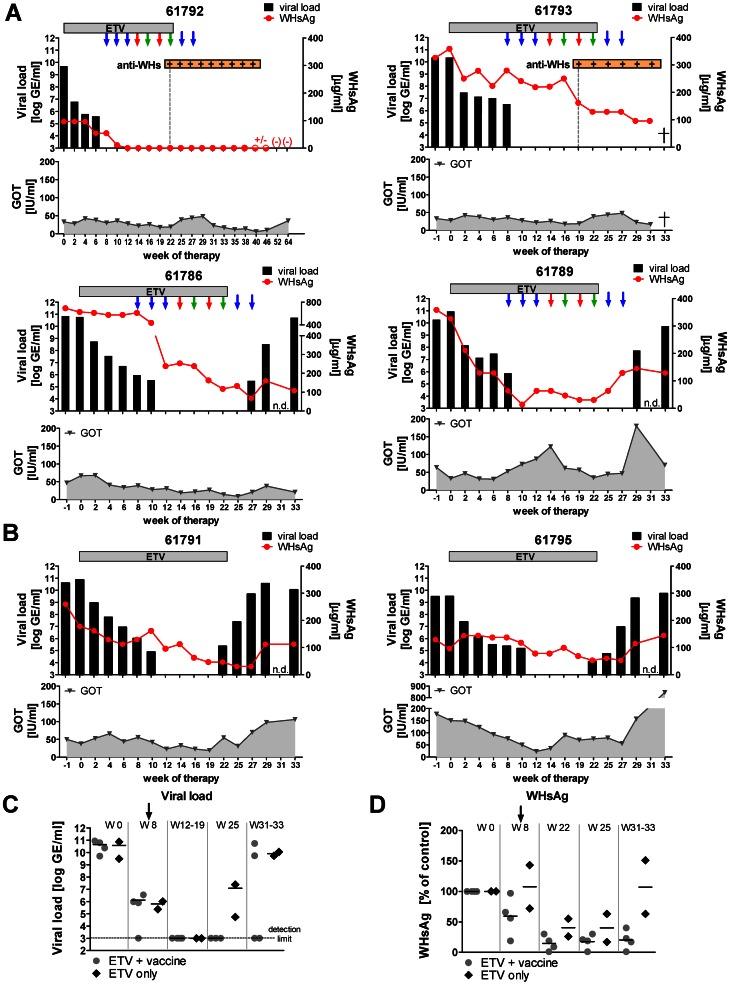
Determination of WHV infection markers in immunized and only entecavir-treated chronic WHV carrier woodchucks. Six chronically WHV-infected woodchucks were treated with ETV for 23 weeks. Four of them (61792, 61793, 61786 and 61789) received subsequently 9 intramuscular immunizations with DNA plasmids, expressing WHcAg and WHsAg (blue arrows), Ad5WHc (red arrows), and Ad35WHc (green arrows) (A). Two animals (number 61791 and 61795) were treated only with ETV and served as controls (B). Summarized comparison of WHV load (C) and WHsAg levels (D) detected in woodchucks receiving immunizations and only ETV-treated controls at representative time points of the therapy. The black arrow represents beginning of the immunization regimen at week 8. The viral DNA was extracted from woodchuck sera and the viral loads were quantified per ml of serum, using real-time PCR analysis. Serum WHsAg concentration was determined by electroimmunodiffusion using rabbit anti-WHs serum. WHsAg-specific antibodies were (anti-WHs) detected in woodchuck sera using protein G coupled to peroxidase. The GOT levels in woodchuck sera were quantified using the standard diagnostic methods. The GOT value above 50 IU/l was considered elevated (GE – genome equivalents; n.d. – not done; † - dead).

The WHV load decreased for approximately 5-logs during the first 8 weeks of the ETV pre-treatment period in all examined woodchucks (Fig. 7A–B). At the time of the first immunization (week 8), no significant difference in the viral loads between the woodchucks from combination therapy group and ETV only treated controls was observed (Fig. 7C). Only woodchuck 61792 showed the viral load below the detection limit at this time point. Between weeks 12 to 19 of therapy all woodchucks remained WHV negative in the blood. Following the decrease in viral load, levels of WHsAg in the sera decreased significantly in all woodchucks during the ETV treatment. The serum GOT initially reached levels below 50 IU/l in the sera of most of the examined woodchucks at weeks 12–14 of the therapy, indicating the reduction of liver inflammation by ETV-mediated decrease of WHV replication (Fig. 7A–B). From the combination therapy group, only woodchuck 61789 showed significant elevation in GOT levels between week 10 and 14 (73–122 IU/ml), indicating a massive cytotoxic T-cells activity in the liver. The GOT levels for other woodchucks from this group slightly increased and were fluctuating around the value of 50 IU/ml. This observation suggests that the therapy induced a gradual elimination of the virus from the liver by the WHV-specific T-cells, without “acute” hepatotoxic effect. Starting from week 16, the constant elevation in serum GOT levels was observed in one of the only ETV-treated control woodchucks: 61795 (75–90 IU/ml). This woodchuck did not show any WHV-specific T-cell response, and these elevated GOT levels might be a symptoms of progressing liver disease. After the end of ETV treatment, the GOT levels rapidly increased in both control WHV carriers and reached the values 106 IU/l in woodchuck 61791 and 845 IU/l in woodchuck 61795 at the end of monitoring period (weeks 31–33). At the same time point, the GOT values in woodchucks that received immunizations (61792, 61793, 61786, and 61789) were considerably lower (12, 17, 21 and 71 IU/l).

As shown in Fig. 7A, the two woodchucks from the combination therapy group (61792 and 61793) were WHV negative until the end of the monitoring period (week 62 and 31, respectively). Moreover, these two woodchucks became anti-WHs positive (week 22 and 19, respectively). Woodchuck 61792 had one of the lowest serum WHsAg levels (96.20 µg/ml) at the beginning of the experiment. The WHsAg became detectable but not quantifiable at week 12 of the therapy and was finally cleared from serum of this woodchuck at week 52. The effect of the combination therapy on the WHsAg in the other woodchuck 61793, which developed anti-WHs antibodies, is difficult to assess due to the short monitoring period. At week 31 the animal had to be euthanized due to serious health problems not related to WHV infection (bacterial infection). Woodchuck 61793 showed over 4-times higher WHsAg levels (424.32 µg/ml) at week 0 than woodchuck 61792. In addition, the reduction in the WHsAg due to the antiviral treatment was not so prominent in woodchuck 61793. After anti-WHs development at week 19, the level of WHsAg dropped in the serum for about 30% within 3 weeks. At the end of the monitoring period woodchuck still showed decreasing tendency in WHsAg levels.

The other woodchucks receiving the immunizations (animals 61786 and 61789) showed rebound of viremia and increased WHsAg levels at the end of the experiment. These woodchucks did not seroconverted to anti-WHs. Nevertheless, our results clearly show that heterologous DNA prime – AdV boost regimen leads to prolonged suppression of WHV replication (5 to 7 weeks), and as a consequence higher decrease in WHsAg in woodchucks 61786 and 61789, compared to only ETV-treated control animals (Fig. 7C). Reappearance of WHV DNA in the serum of the control woodchucks was seen at the end of ETV therapy (week 22). The woodchuck 61786 and 61789 showed a rebound of the viremia at week 27 and 29, respectively. As shown in Fig. 7D, these animals showed a more prominent decrease in the WHsAg levels at week 22 [81% and 91%] than control woodchucks 61791 and 61795 [74% and 45%]. At the end of monitoring period, woodchucks from combination therapy group showed 23%, 17%, and 40% of the baseline WHsAg levels (91793, 61786, 61789, respectively; the very low amount of WHsAg was not quantifiable for woodchuck 61792 at that time), whereas controls had 63% and 151% of baseline WHsAg level.

**Figure 8:**
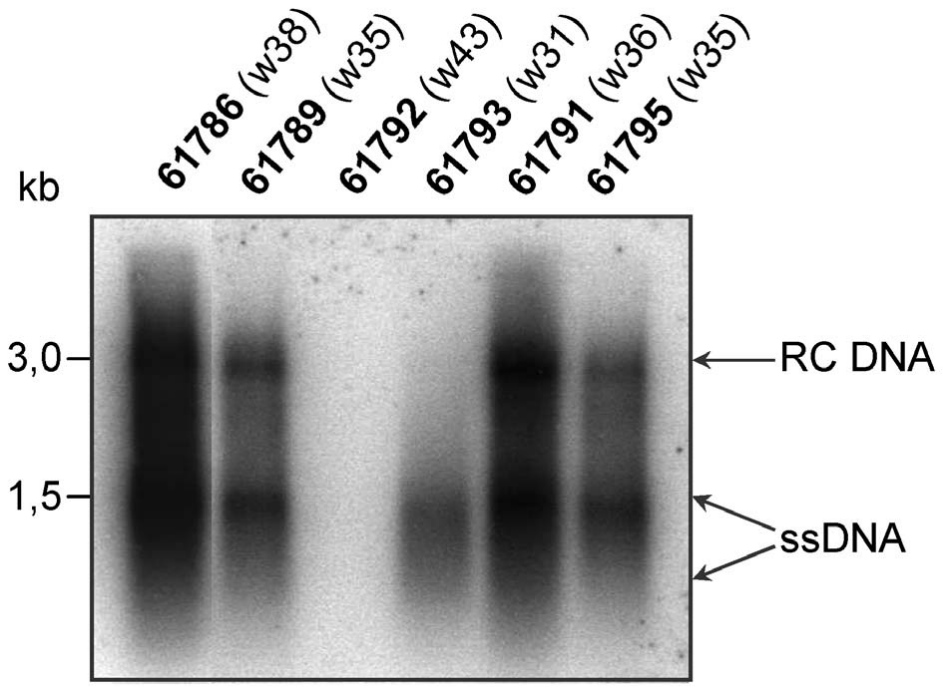
Determination of WHV replication in the livers of chronically WHV-infected woodchucks. Southern blot analysis was performed on the DNA obtained from the liver samples collected post-mortem or by liver biopsy from chronically WHV-infected woodchucks at given time points (W – week). Woodchucks number 61786, 61789, 61792 and 61793 were treated with combination therapy. Woodchucks 61791 and 61795 were treated only with ETV and served as controls. Total amount of 10 µg of isolated DNA was electrophoresed into agarose gel and then transferred onto nylon membrane. WHV replicative intermediates were detected by hybridization with [^32^P] labelled full-length WHV strain 8 genome as a probe. The arrows indicate the relaxed circular WHV DNA (RC DNA; 3,0 kb) and single-stranded WHV DNA (ssDNA; approximately 1,5 kb).

In addition, we evaluated the replication of WHV in the liver samples collected post-mortem or through liver biopsy. Figure 8 shows the Southern blotting of WHV replicative intermediates, corresponding to the single-stranded DNA (ssDNA) and relaxed circular DNA (RC DNA). The two woodchucks which were WHV DNA negative in the serum and developed anti-WHs antibodies (61792 and 61793) showed no or very low WHV replication in the liver. The other WHV chronic carriers showed comparable levels of WHV replication at the time points of sacrifice.

## Discussion

Studies in preclinical models of HBV infection such as woodchucks and chimpanzees as well as patients underline the important role of HBV-specific T-cell response as a leading factor of viral clearance [3], [21], [23]–[26]. In the presented study, we demonstrated that the heterologous DNA prime – recombinant AdV boost immunization is able to induce an effective virus-specific T-cell response to WHV antigens and efficiently suppress the viral replication in WHV transgenic mice and chronically WHV-infected woodchucks.

Consistent to our previous studies in mice [27] the heterologous DNA prime – AdV boost regimens proved to be superior to DNA-only regimen also in WHV Tg mice. Mice immunized in DNA prime - Ad5WHc boost manner developed significantly higher levels of anti-WHc antibodies, and significantly more WHcAg-specific CD8^+^ T-cells in comparison to the group of animals immunized with DNA only. Interestingly, the fourth immunization with Ad35WHc (DNA-Ad5-Ad35WHc) was correlated with the development of anti-WHs antibodies. This experiment shows, that core-specific T helper response is able to prime WHsAg-specific B cells, consistent with the concept of “intermolecular help” [34]. At the same time, the magnitude of CD8^+^ T-cell response did not increase after Ad35WHc boost and remained comparable to mice that received only Ad5WHc. As these mice show inbred tolerance to WHV proteins, the maximal possible level of WHV-specific T-cell responses that could be induced by vaccination was probably achieved already after single Ad5WHc boost. However, WHV-specific antibody could be further expanded, as our vaccines were specifically designed to prime vigorous T-cell responses, but were quite poor inducers of humoral immune response (especially DNA vaccine). Even though the strength of induced CD8^+^ T-cell response in WHV Tg mice was approximately 10 times lower than that detected in naïve mice [27], the immunization with optimized vaccines was able to significantly reduce the WHV load in these mice. This effect was clearly due to potent and functional WHcAg-specific T-cell responses in the liver, the major compartment of WHV replication. The pronounced suppression in WHV replication was observed in DNA-Ad5WHc or DNA-Ad5-Ad35WHc vaccination groups, in which more than 70% of mice had undetectable viral loads at the end of the experiment.

It was previously demonstrated that combination of ETV treatment and prime –boost vaccination with DNA and recombinant fowlpoxvirus expressing core and surface antigens of duck hepatitis B virus (DHBV) prevented the development of persistent infection in ducks [38]. Here, we examined the efficacy of the new DNA prime – AdV boost vaccination in combination with entecavir for the treatment of already established chronic hepadnaviral infection (more than 1 year) in woodchucks. There is an open scientific debate suggesting that multiple and frequent administration of the vaccine may be advisable in treatment of chronic hepatitis [39], [40]. Chronically infected individuals usually exhibit the immune tolerance to targeted antigens. Therefore, the effect of the immunization results in much weaker response that and rarely reaches the level that would be expected in naïve patients or animals. Our immunization regimen based on 2–3 week time intervals between the immunizations enhanced the antiviral effect of ETV monotherapy and induced improved WHV-specific immune responses, resulting in the long term suppression of viral replication, and subsequently viral clearance in some animals.

Previous reports indicate that combination of antiviral treatment and therapeutic vaccination may partially restore WHV-specific T-cell responses in chronic WHV carriers [30], [32]. In our study, WHV-specific helper and cytotoxic T-cell responses were detected in PBMCs of all four chronically WHV-infected woodchucks that received the therapeutic vaccine. This outcome was clearly an effect of the improved therapeutic DNA prime–recombinant adenovirus boost immunization strategy, since ETV-only treatment did not induce sustained and significant T-cell responses in the control animals. The WHsAg-specific proliferative responses were predominantly directed against the peptides s224-239 and s252-267. The position of the epitope s224-239 overlapped with the peptide s226-245, preferentially recognized in WHV chronic carriers after clevudine/WHsAg combination therapy [30]. Moreover, several WHcAg-derived T_H_ epitopes identified in woodchucks with acute self-limited WHV infection [3] were recognized in WHV carriers.

Following the appearance of T helper cells, WHcAg- and WHsAg-specific CTLs were detectable in all woodchucks that received combination therapy. Only brief CTL responses were detected shortly after the beginning of ETV treatment. Those findings are consistent with the data obtained from chronic HBV patients treated with nucleoside analogues [28], [29]. The appearance of sustained WHcAg-specific CTL response after the vaccinations was observed after Ad5WHc and Ad35WHc administration. All four woodchucks receiving vaccinations demonstrated significant WHcAg-specific CTL responses, whereas the WHsAg-specific CTL responses were not as prominent and appeared only transiently. Nevertheless, the contribution of these WHsAg-specific T-cell responses to overall therapeutic effect cannot be decisively assessed. Due to the high costs of woodchucks and long experimental periods, we could not yet provide the detailed answer whether both components are required for a successful therapeutic vaccination. This question needs to be addressed in the future studies on the larger number of woodchucks.

The crucial criteria for resolution of HBV infection in humans are reduction of HBV load below the detection limit, loss of HBsAg and seroconversion to anti-HBs [41]. Colonno *et al.* reported that long-term ETV treatment (over 1 to 3 years) may be associated with partial control of WHV replication post-treatment [33]. These woodchucks showed very low levels of viral DNA, however, none of them developed anti-WHs antibodies. In addition to this study, we currently performed two other independent studies (that are to be published soon), including in total 8 chronic WHV carriers treated only with ETV. The results of these studies are consistent with the findings described here. All woodchucks showed the rebound of WHV replication shortly after the end of ETV treatment (*our unpublished results*).

Our results demonstrate that the two immunized woodchucks (61792 and 61793) were WHV negative until the end of the monitoring period and developed anti-WHs as a proof of a sustained antiviral response. Interestingly, these therapeutic effects seem to be associated with induction of WHV-specific T-cell response by vaccination. These two woodchucks showed the earliest, strongest and the most sustained WHV-specific T-cell responses of all tested animals. In addition, woodchuck 61792 was negative for WHV replicative intermediates in the liver and cleared WHsAg from the blood. The process of the WHsAg clearance in woodchuck 61792 took more than 52 weeks. Since the WHV genome is integrated within the woodchuck genome [42] and a distinct pathway of surface antigen secretion was recently demonstrated [43] we assume that WHsAg clearance was achieved through elimination of the WHV-infected hepatocytes by virus-specific cytotoxic T-cells as well as hepatocyte turnover process. Woodchuck 61793 had to be prematurely euthanized due to a bacterial infection, and therefore was monitored for a much shorter period (31 weeks) than woodchuck 61792 (64 weeks). At the time point of sacrifice, WHV ssDNA replicative intermediate was still present in the liver of woodchuck 61793, implying that the WHV replication could be not completely suppressed in this animal. Nevertheless, residual HBV replication in the liver can be also observed in the patients who recovered from acute hepatitis B, despite the presence of anti-HBs. These patients are not viremic due to the high neutralizing anti-HBs antibody concentration, which prevent the reinfection of the uninfected hepatocytes [7], [44]. Woodchuck 61793 developed anti-WHs, and the WHsAg load was continuously decreasing at the end of the monitoring period in this woodchuck. As the clearance of WHsAg in woodchuck 61792, with approximately 4-times lower baseline level took a considerate amount of time (52 weeks), this decreasing tendency may indicate that long-term control of WHV infection in woodchuck 61793 was achieved.

Some studies in chronically HBV-infected patients suggest that a low baseline HBsAg levels are associated with control of replication post therapy with nucleos(t)ide analogues [45], [46]. However, no information about the influence of HBsAg levels on the effects of immunotherapeutic approaches is available in patients yet. In our previous study, using lamivudine treatment together with WHsAg/anti-WHs immune complexes vaccination, we observed the induction of anti-WHs antibodies predominantly in the woodchucks with high baseline WHsAg loads [32]. Nevertheless, these antibody responses were not sustained. In this study, we could demonstrate that the baseline of WHsAg was a poor predictor of the overall therapeutic effect. The WHsAg seroconversion was achieved in two animals: one with of the lowest, and one with the second highest WHsAg baseline values.

It can be concluded that by (1) the addition of a potent antiviral drug (entecavir) (2) using the improved vectors for therapeutic vaccination, (3) and the prime-boost vaccination protocol, a novel and effective strategy in treatment of chronic hepadnaviral infections was obtained in the preclinical model. These findings may imply to perform the new clinical trials of therapeutic vaccination, using prime-boost regimens with DNA vaccines and recombinant viral vectors (AdV or modified vaccinia ankara (MVA)) in chronically HBV-infected patients.

## Materials and Methods

### Ethical statement

All animal experiments were conducted in accordance with the *Guide for the Care and Use of Laboratory Animals* and were approved by the local Animal Care and Use Committee (Animal Care Center, University of Duisburg-Essen, Essen, Germany and the district government of Düsseldorf, Germany; permission numbers G1109/10 and G1117/10). The experiments were performed under isofluran or ketamine-xylazine anesthesia, and all efforts were made to minimize suffering.

### Laboratory animals

WHV transgenic (Tg) mice lineages carrying WHV wild-type (strain 1217) and a mutated transgenome lacking the L-, M- and S-WHsAg (strain 1281) were created on C57BL/6 background (genotype H-2b/b) and previously characterized [*Meng et al., manuscript attached*]. Wild-trapped chronically WHV-infected woodchucks were purchased from North Eastern Wildlife (Harrison, ID). Laboratory animals were maintained according to the guidelines of the animal facility at the University Hospital Essen.

### Vaccines

The construction of pCGWHc plasmid and recombinant adenoviral vectors serotype 5 (Ad5WHc) and chimeric Ad5 displaying Ad35 fiber (Ad35WHc) expressing WHcAg was described previously [27]. Briefly, the WHV strain 8 WHcAg gene was obtained from WHcAg-encoding plasmid pWHcIm [47] and introduced between a β-globin intron sequence and polyadenylation signal of pCG vector [48]. The adenoviral vectors expressing WHcAg (Ad5WHc and Ad35WHc) were constructed using the AdEasy system and vectors pShuttle, pAdEasy-1 and pAdEasy-1/F35 (Qbiogene, Carlsbad, CA). Generation of WHsAg-expressing pWHsIm plasmid was described earlier [27], [47].

### Immunization of WHV transgenic mice with heterologous DNA prime – AdV boost regimen

Ten to twelve weeks old sex-matched groups of mice were pretreated by intramuscular injection of 50 µl of cardiotoxin (10 µM in PBS; Latoxan, Valence, France) into *Tibialis anterior* muscle one week before the plasmid immunization. Animals were then intramuscularly vaccinated twice with 100 µg of pCGWHc (50 µg per muscle) at two weeks interval. Four weeks after the second DNA immunization groups of mice were immunized with 2×10^9^ PFU of Ad5WHc or 2×10^9^ PFU of Ad35WHc or 100 µg pCGWHc as a reference, according to the protocol described previously [27]. The group of mice immunized twice with pCGWHc in combination with Ad5WHc was boosted for a second time with 2×10^9^ PFU of Ad35WHc. The vaccination was performed four weeks after Ad5WHc immunization. Mice of the control group were immunized twice with 100 µg of ‘empty’ pCG and boosted with 2×10^9^ PFU of Ad5 expressing green fluorescent protein (GFP; kindly provided by Dr. W. Bayer, Institute of Virology, University Hospital of Essen). Mice were sacrificed two weeks after the last immunization.

### Therapeutic vaccination of chronically WHV-infected woodchucks in combination with entecavir (ETV) treatment

Six chronically WHV-infected woodchucks (number: 61786, 61789, 61791, 61792, 61793 and 61795) were treated for 23 weeks with the nucleoside analogue entecavir (ETV, Bristol-Myers Squibb, New York, NY). Initially, the drug was administered for 12 weeks at dosage 1.4 mg per week by osmotic pumps (DURECT, Cupertino, CA) implanted surgically under the skin of the animals. From week 8 to 23 of the therapy subcutaneous injections of 1 mg of ETV were performed twice a week. At week 7, four of the six ETV-treated animals (number: 61786, 61789, 61792, and 61793) were pretreated by intramuscular injection of 250 µl of cardiotoxin (10 µM in PBS; Latoxan) into *Tibialis anterior* muscle. Starting from week 8 the animals received subsequently 9 intramuscular immunizations with: DNA plasmids, expressing WHcAg (pCGWHc) and WHsAg (pWHsIm) at weeks 8, 10, 12, 25 and 27; Ad5WHc+pWHsIm at weeks 14 and 19; Ad35WHc+pWHsIm at week 16 and 22 of the therapy, as shown in Fig. 4. For the vaccination 0,5 mg of plasmids and 1×10^11^ PFU (plague forming units) of Ad5WHc or Ad35WHc was used. Two animals (number: 61791, and 61795) treated only with ETV served as controls.

### Cell culture

Murine lymphocytes and woodchuck PBMCs were cultured in RPMI medium (Invitrogen/Gibco, Karlsruhe, Germany) and AIM-V medium (Invitrogen/Gibco), respectively. Cell culture media were supplemented with 10% heat-inactivated fetal bovine serum (FBS; Biochrom AG, Berlin, Germany) and 10 U/ml penicillin-streptomycin (PAA Laboratories, Pasching, Austria). Cells were maintained in a humidified 5% CO_2_ atmosphere at 37°C.

### Isolation of splenic and hepatic lymphocytes from mouse

Preparation of a single-cell suspensions of murine splenocytes was performed according to the procedure described previously [37]. Up to 1×10^6^ of isolated splenocytes per well were plated in 96-well plates in 200 µl of cell culture medium. Hepatic lymphocytes were isolated from the liver using published methods [49], [50] with some modifications. Briefly, livers were perfused with prewarmed PBS (to flush blood from the hepatic vasculature) and were forced through a 70 µm nylon cell strainer (BD Falcon, Franklin Lakes, NJ). After washing, cell pellets were suspended in 5 ml of prewarmed enzyme solution, containing 0,05% Collagenase type II (Sigma) and 500 U/ml DNAse type I (Sigma) in Ca^2+^/Mg^2+^-free HBSS supplemented with 10% FBS, and digested for 40 min at 37°C. Cells were then layered on 40% Percoll solution (Sigma) in RPMI 1640 supplemented with 10 U/ml penicillin-streptomycin for density separation, and centrifuged at 300× g for 17 minutes at 4°C without brakes. Cell pellets were washed and suspended in 2 ml of Buffer EL (Qiagen, Hilden, Germany) to lyse red blood cells. Cell yields and viabilities were determined by trypan blue exclusion microscopy.

### *In vitro* stimulation of murine lymphocytes

Murine lymphocytes were stimulated 6 hours or 7 days (in the presence of 10 U/ml of recombinant murine IL-2; Roche) with the previously identified WHcAg-derived CD8^+^ epitope c13-23 (YQLLNFLPL) and CD4^+^ epitope c131-145 (PYRPPNAPILSTLPE) [27], added to a final concentration of 2 µg/ml. Unstimulated cells and cells stimulated with CMV-derived peptide (YILEETSVM) served as negative controls. Prior to intracellular cytokine staining, cells were cultured for 5–6 hours in the presence of 1 µg/ml of α-CD28 antibody (clone 37.51; BD Pharmingen, Heidelberg, Germany) and 5 µg/ml of Brefeldin A (Sigma-Aldrich).

### Cell surface and intracellular cytokine staining of murine splenic and hepatic lymphocytes

Cell surface staining was performed using the anti-CD8 (clone 56.6-7; BD Pharmingen) and anti-CD4 (clone L3T4; BD Pharmingen) T-cell antibodies. Staining of CD107a molecule (monoclonal anti-mouse CD107a antibody, clone GB12, dilution 1∶200; BD Pharmingen) was performed during 5 h restimulation of the splenocytes. Dead cells were excluded from analyses using 7-aminoactinomycin D (7AAD) (Beckton Dickinson, Heidelberg, Germany). Intracellular cytokine stainings were performed as described elsewhere [51] with the following antibodies: anti-IFN-γ (clone XMG1.2; BD Pharmingen), anti-TNF-α (clone MP6-XT22; eBioscience, Hatfield, United Kingdom) and anti-IL-2 (clone JES6-5H4, eBioscience). Data were acquired on FACS-Calibur or LSR II flow cytometers (Becton Dickinson, Heidelberg, Germany) from 150 000–300 000 lymphocyte-gated events per sample. Analyses were performed using FlowJo software (Tree Star, Ashland, OR).

### Detection of WHcAg-specific CD8^+^ T-cells

WHcAg-specific CD8^+^ T-cells were detected using soluble DimerX H-2D^b^:Ig fusion protein technology (BD Pharmingen) according to the manufacturer's instructions. The H-2D^b^ dimer consists of two extracellular major histocompatibility complex class I (MHC-I) H-2D^b^ domains that are fused to variable regions of mouse IgG_1_. Briefly, 0,8 µg of dimer per sample was loaded with 2,4 µg of H-2D^b^-restricted WHcAg-derived CD8^+^ epitope c13-23 overnight in 37°C. Freshly isolated splenic lymphocytes were pre-treated with anti-CD16/anti-CD32 antibodies (Fc-Block, clone 2.4G2; BD Pharmingen) diluted 1∶200 for 30 min in 4°C. Next, cells were incubated with anti-CD8^+^ antibody and 4 µl of peptide-loaded dimer per sample for 1 h in 4°C. As control mouse IgG_1_ isotype control antibody (clone MOPC-21/P3; eBioscience) was used. The detection of dimer- CD8^+^ T-cell complexes was performed by staining with secondary anti-mouse IgG1 antibody (clone: A85-1, BD Pharmingen) diluted 1∶200 (30 min, 4°C).

### CD107a degranulation assay of woodchuck PBMCs

Woodchuck PBMCs were separated by Ficoll density gradient centrifugation and cultivated as described previously [37]. For stimulation, the previously identified WHcAg-derived epitope c96-110 (KVRQSLWFHLSCLTF) and a WHsAg-derived epitope s220-234 (AGLQVVYFLWTKILT) [37] were added to a final concentration of 2 µg/ml per peptide. Unstimulated cells and cells stimulated with CMV-derived peptide (YILEETSVM) served as negative controls. After 3 days of *in vitro* stimulation, cells were re-stimulated and stained for CD107a molecule with anti-mouse CD107a FITC-conjugated antibody (clone GB12, dilution 1∶100; BD Pharmingen) as described previously [37]. For CD4 detection anti-human CD4 allophycocyanin-conjugated antibody (clone L200; BD Pharmingen) was used. Dead cells were excluded from analyses using 7AAD.

### Proliferation of woodchuck PBMCs

Antigen-specific proliferation of woodchuck PBMCs was determined by 2[^3^H]adenine-based assay as described previously [47]. Briefly, 5×10^4^ PBMCs were stimulated with a synthetic peptides added to a final concentration of 5 µg/ml for 5 days. For stimulation a panel of 10 WHcAg-derived peptides (EMC microcollections, Tübingen, Germany) and 16 WHsAg-derived peptides containing the known woodchuck T_H_ epitopes was used (see supplementary Table S2 and S3). Unstimulated cells and cells stimulated with CMV-derived peptide (YILEETSVM) served as a negative control. Afterwards, cells were labelled with 1 µCi of 2[^3^H]-adenine (Hartmann Analytic, Braunschweig, Germany) for 16 h and collected using a cell harvester (Perkin Elmer, Waltham, MA). Results for triplicate cultures are presented as a mean stimulation index (SI) [(mean total absorption for stimulated PBMCs)/(mean total absorption for unstimulated control)]. A SI≥3.0 was considered significant.

### Serology

Murine WHcAg-specific IgG, IgG_1_ and IgG_2a_ as well as woodchuck anti-WHc and anti-WHs antibodies were detected by enzyme-linked immunosorbent assay (ELISA) as described previously [47], [52].

### Quantification of WHV DNA in the serum

WHV DNA was quantified by real-time PCR using Platinum SYBR Green Kit (Invitrogen) as described previously [37].

### Analysis of WHV replication intermediates in liver tissues

Total DNA from liver samples of chronically WHV-infected woodchucks was extracted using the QIAamp Tissue Kit (Qiagen, Hilden, Germany) according to the manufacturer's instructions. Total amount of 10 µg of isolated DNA was electrophoresed into agarose gel, then transferred onto Amersham Hybond-N+ positively charged nylon membrane (GE Healthcare, Little Chalfont, United Kingdom) using Vaccum Blotter 785 (Bio-Rad Laboratories, München, Germany). WHV replication intermediates were analyzed by Southern blot hybridization with a full length WHV8 genome as probe using standard procedure [53], [54]. Briefly, the radioactive labelling of the probe was performed using DecaLabel DNA Labelling Kit (Fermentas, St.Leon-Rot, Germany), 50 ng of plasmid and [^32^P]-dCTPs, according to the manufacturer's protocol. The hybridization was performed overnight at 65°C with the probe in RapidHyb Buffer (GE Healthcare, Little Chalfont, United Kingdom). After washing and drying, the membranes were exposed overnight onto the Cyclon's screens (Packard, Meriden, CT, USA). The quantitative analysis of the signals on the blots was performed using a Cyclon Phospho-Imager (Packard, Meriden, CT, USA).

### Determination of WHsAg concentration in woodchuck sera

Serum WHsAg concentration was determined by electroimmunodiffusion in a similar way as described for HBsAg [32], [55]. Glass slides (3×2 inches) were coated with 6 ml of 0.6% agarose, containing 70 µl of rabbit polyclonal anti-WHs anti-serum per ml. Woodchuck serum samples were diluted 1∶10 in fetal calf serum. The volume of 10 µl of the diluted samples was applied in 3 mm holes, and run for 12 hours at 4 mA per slide. The length of the precipitation arc was converted in µg WHsAg/ml using a calibration curve and highly purified WHsAg from chronic WHV-infected woodchucks as reference antigen. The concentration of purified WHsAg was measured by UV spectrophotometry at 280 nm assuming an OD of 5.1 for 1 mg/mL [56]. The coefficient of variation of the assay was approximately 10%.

### Evaluation of GOT levels

The glutamic oxaloacetic transaminase (GOT; also known as aspartate transaminase, AST) activity in the serum was quantified according to standard clinical diagnostic procedures at the Central Laboratory of University Hospital Essen. The values above 50 IU (international units) per millilitre were considered as elevated.

### Statistical analysis

Statistical analyses were performed using GraphPad Prism version 5 (GraphPad Software Inc., San Diego, CA). Statistical differences were analyzed by one-way analysis of variance test using Newman-Keuls multiple comparison post-test. The evaluation of the statistical differences between the viral loads in WHV transgenic mice was performed by The Wilcoxon signed rank test. The *P*-values<0.05 were considered significant.

## Supporting Information

Figure S1 Comparison of WHV-specific antibodies profiles detected in wild-type WHV (1217) and WHsAg-lacking (1281) transgenic mice. Mice were primed two times by immunization with the pCGWHc plasmid and four weeks later a first boosting immunization with Ad5WHc was performed. Mice were boosted for a second time with Ad35WHc. WHcAg-specific IgG, IgG_2a_, IgG_1_ or WHsAg-specific IgG antibodies were detected in sera collected two weeks after the last, boosting immunization (serum dilution 1∶500). Asterisks mark the significant difference (***<0.0005; *ns* – not significant).
(TIFF)

Figure S2 Representative dot-plots of CD107a^+^ degranulation responses detected in treated WHV chronic carriers. Woodchucks number 61786, 61789, 61792 and 61793 were treated with combination therapy (ETV+ vaccine). Woodchucks 61791 and 61795 were treated only with ETV and served as controls. PBMCs were expanded *in vitro* for 3 days with WHcAg-derived epitope c96-110 or WHsAg-derived epitope s220-234. Unstimulated cells and cells stimulated with unrelated CMV-derived peptide served as a negative controls. Presented values shows the percentage of CD107a^+^ CD3^+^ CD4^−^ T-cells in the CD3^+^ CD4^−^ T-cell population.
(TIFF)

Table S1 Positive WHsAg- and WHcAg-specific lymphoproliferative responses (Stimulation index ≥3,0) detected in chronically WHV-infected woodchucks during the therapy.
(DOC)

Table S2 Amino acid sequence of WHcAg-derived peptides used for *in vitro* stimulation of woodchuck lymphocytes (Proliferation assay).
(DOC)

Table S3 Amino acid sequence of WHsAg-derived peptides used for *in vitro* stimulation of woodchuck lymphocytes (Proliferation assay).
(DOC)

## References

[1] Ratnam D, Dev A, Nguyen T, Sundararajan V, Harley H, et al (2011) The Efficacy and Tolerability of Pegylated Interferon-alpha-2a in Chronic Hepatitis B: A Multicenter Clinical Experience. J Gastroenterol Hepatol 27: 1447–53. 10.1111/j.1440-1746.2011.07051.x 22168789

[2] Lau GK, Piratvisuth T, Luo KX, Marcellin P, Thongsawat S, et al (2005) Peginterferon Alfa-2a, lamivudine, and the combination for HBeAg-positive chronic hepatitis B. N Engl J Med 352: 2682–2695. 15987917 10.1056/NEJMoa043470

[3] Menne S, Maschke J, Lu M, Grosse-Wilde H, Roggendorf M (1998) T-Cell response to woodchuck hepatitis virus (WHV) antigens during acute self-limited WHV infection and convalescence and after viral challenge. J Virol 72: 6083–6091. 9621072 10.1128/jvi.72.7.6083-6091.1998 PMC110414

[4] Webster GJ, Reignat S, Maini MK, Whalley SA, Ogg GS, et al (2000) Incubation phase of acute hepatitis B in man: dynamic of cellular immune mechanisms. Hepatology 32: 1117–1124. 11050064 10.1053/jhep.2000.19324

[5] Jung MC, Spengler U, Schraut W, Hoffmann R, Zachoval R, et al (1991) Hepatitis B virus antigen-specific T-cell activation in patients with acute and chronic hepatitis B. J Hepatol 13: 310–317. 1808224 10.1016/0168-8278(91)90074-l

[6] Penna A, Chisari FV, Bertoletti A, Missale G, Fowler P, et al (1991) Cytotoxic T lymphocytes recognize an HLA-A2-restricted epitope within the hepatitis B virus nucleocapsid antigen. J Exp Med 174: 1565–1570. 1720813 10.1084/jem.174.6.1565 PMC2119048

[7] Rehermann B, Nascimbeni M (2005) Immunology of hepatitis B virus and hepatitis C virus infection. Nat Rev Immunol 5: 215–229. 15738952 10.1038/nri1573

[8] Yang PL, Althage A, Chung J, Maier H, Wieland S, et al (2010) Immune effectors required for hepatitis B virus clearance. Proc Natl Acad Sci U S A 107: 798–802. 20080755 10.1073/pnas.0913498107 PMC2818933

[9] Couillin I, Pol S, Mancini M, Driss F, Brechot C, et al (1999) Specific vaccine therapy in chronic hepatitis B: induction of T cell proliferative responses specific for envelope antigens. J Infect Dis 180: 15–26. 10353856 10.1086/314828

[10] Dahmen A, Herzog-Hauff S, Bocher WO, Galle PR, Lohr HF (2002) Clinical and immunological efficacy of intradermal vaccine plus lamivudine with or without interleukin-2 in patients with chronic hepatitis B. J Med Virol 66: 452–460. 11857521 10.1002/jmv.2165

[11] Dikici B, Bosnak M, Ucmak H, Dagli A, Ece A, et al (2003) Failure of therapeutic vaccination using hepatitis B surface antigen vaccine in the immunotolerant phase of children with chronic hepatitis B infection. J Gastroenterol Hepatol 18: 218–222. 12542609 10.1046/j.1440-1746.2003.02950.x

[12] Horiike N, Fazle Akbar SM, Michitaka K, Joukou K, Yamamoto K, et al (2005) In vivo immunization by vaccine therapy following virus suppression by lamivudine: a novel approach for treating patients with chronic hepatitis B. J Clin Virol 32: 156–161. 15653419 10.1016/j.jcv.2004.07.004

[13] Jung MC, Gruner N, Zachoval R, Schraut W, Gerlach T, et al (2002) Immunological monitoring during therapeutic vaccination as a prerequisite for the design of new effective therapies: induction of a vaccine-specific CD4+ T-cell proliferative response in chronic hepatitis B carriers. Vaccine 20: 3598–3612. 12297407 10.1016/s0264-410x(02)00309-2

[14] Pol S, Driss F, Michel ML, Nalpas B, Berthelot P, et al (1994) Specific vaccine therapy in chronic hepatitis B infection. Lancet 344: 342. 10.1016/s0140-6736(94)91384-6 7914291

[15] Pol S, Nalpas B, Driss F, Michel ML, Tiollais P, et al (2001) Efficacy and limitations of a specific immunotherapy in chronic hepatitis B. J Hepatol 34: 917–921. 11451177 10.1016/s0168-8278(01)00028-9

[16] Ren F, Hino K, Yamaguchi Y, Funatsuki K, Hayashi A, et al (2003) Cytokine-dependent anti-viral role of CD4-positive T cells in therapeutic vaccination against chronic hepatitis B viral infection. J Med Virol 71: 376–384. 12966542 10.1002/jmv.10509

[17] Safadi R, Israeli E, Papo O, Shibolet O, Melhem A, et al (2003) Treatment of chronic hepatitis B virus infection via oral immune regulation toward hepatitis B virus proteins. Am J Gastroenterol 98: 2505–2515. 14638356 10.1111/j.1572-0241.2003.07700.x

[18] Vandepapeliere P, Lau GK, Leroux-Roels G, Horsmans Y, Gane E, et al (2007) Therapeutic vaccination of chronic hepatitis B patients with virus suppression by antiviral therapy: a randomized, controlled study of co-administration of HBsAg/AS02 candidate vaccine and lamivudine. Vaccine 25: 8585–8597. 18031872 10.1016/j.vaccine.2007.09.072

[19] Yalcin K, Acar M, Degertekin H (2003) Specific hepatitis B vaccine therapy in inactive HBsAg carriers: a randomized controlled trial. Infection 31: 221–225. 14562945 10.1007/s15010-003-3187-1

[20] Mancini-Bourgine M, Fontaine H, Scott-Algara D, Pol S, Brechot C, et al (2004) Induction or expansion of T-cell responses by a hepatitis B DNA vaccine administered to chronic HBV carriers. Hepatology 40: 874–882. 15382173 10.1002/hep.20408

[21] Ferrari C, Penna A, Bertoletti A, Valli A, Antoni AD, et al (1990) Cellular immune response to hepatitis B virus-encoded antigens in acute and chronic hepatitis B virus infection. J Immunol 145: 3442–3449. 2230128

[22] Guidotti LG, Rochford R, Chung J, Shapiro M, Purcell R, et al (1999) Viral clearance without destruction of infected cells during acute HBV infection. Science 284: 825–829. 10221919 10.1126/science.284.5415.825

[23] Maini MK, Boni C, Lee CK, Larrubia JR, Reignat S, et al (2000) The role of virus-specific CD8(+) cells in liver damage and viral control during persistent hepatitis B virus infection. J Exp Med 191: 1269–1280. 10770795 10.1084/jem.191.8.1269 PMC2193131

[24] Penna A, Artini M, Cavalli A, Levrero M, Bertoletti A, et al (1996) Long-lasting memory T cell responses following self-limited acute hepatitis B. J Clin Invest 98: 1185–1194. 8787682 10.1172/JCI118902 PMC507541

[25] Penna A, Del Prete G, Cavalli A, Bertoletti A, D'Elios MM, et al (1997) Predominant T-helper 1 cytokine profile of hepatitis B virus nucleocapsid-specific T cells in acute self-limited hepatitis B. Hepatology 25: 1022–1027. 9096614 10.1002/hep.510250438

[26] Thimme R, Wieland S, Steiger C, Ghrayeb J, Reimann KA, et al (2003) CD8(+) T cells mediate viral clearance and disease pathogenesis during acute hepatitis B virus infection. J Virol 77: 68–76. 12477811 10.1128/JVI.77.1.68-76.2003 PMC140637

[27] Kosinska AD, Johrden L, Zhang E, Fiedler M, Mayer A, et al (2012) DNA Prime-Adenovirus Boost Immunization Induces a Vigorous and Multifunctional T-Cell Response against Hepadnaviral Proteins in the Mouse and Woodchuck Model. J Virol 86: 9297–9310. 22718818 10.1128/JVI.00506-12 PMC3416123

[28] Boni C, Penna A, Bertoletti A, Lamonaca V, Rapti I, et al (2003) Transient restoration of anti-viral T cell responses induced by lamivudine therapy in chronic hepatitis B. J Hepatol 39: 595–605. 12971971 10.1016/s0168-8278(03)00292-7

[29] Boni C, Penna A, Ogg GS, Bertoletti A, Pilli M, et al (2001) Lamivudine treatment can overcome cytotoxic T-cell hyporesponsiveness in chronic hepatitis B: new perspectives for immune therapy. Hepatology 33: 963–971. 11283861 10.1053/jhep.2001.23045

[30] Menne S, Roneker CA, Tennant BC, Korba BE, Gerin JL, et al (2002) Immunogenic effects of woodchuck hepatitis virus surface antigen vaccine in combination with antiviral therapy: breaking of humoral and cellular immune tolerance in chronic woodchuck hepatitis virus infection. Intervirology 45: 237–250. 12566706 10.1159/000067914

[31] Hervas-Stubbs S, Lasarte JJ, Sarobe P, Vivas I, Condreay L, et al (2001) T-helper cell response to woodchuck hepatitis virus antigens after therapeutic vaccination of chronically-infected animals treated with lamivudine. J Hepatol 35: 105–111. 11495027 10.1016/s0168-8278(01)00063-0

[32] Lu M, Yao X, Xu Y, Lorenz H, Dahmen U, et al (2008) Combination of an antiviral drug and immunomodulation against hepadnaviral infection in the woodchuck model. J Virol 82: 2598–2603. 18160442 10.1128/JVI.01613-07 PMC2258919

[33] Colonno RJ, Genovesi EV, Medina I, Lamb L, Durham SK, et al (2001) Long-term entecavir treatment results in sustained antiviral efficacy and prolonged life span in the woodchuck model of chronic hepatitis infection. J Infect Dis 184: 1236–1245. 11679911 10.1086/324003

[34] Milich DR, McLachlan A, Thornton GB, Hughes JL (1987) Antibody production to the nucleocapsid and envelope of the hepatitis B virus primed by a single synthetic T cell site. Nature 329: 547–549. 2443856 10.1038/329547a0

[35] Betts MR, Brenchley JM, Price DA, De Rosa SC, Douek DC, et al (2003) Sensitive and viable identification of antigen-specific CD8+ T cells by a flow cytometric assay for degranulation. J Immunol Methods 281: 65–78. 14580882 10.1016/s0022-1759(03)00265-5

[36] Rubio V, Stuge TB, Singh N, Betts MR, Weber JS, et al (2003) Ex vivo identification, isolation and analysis of tumor-cytolytic T cells. Nat Med 9: 1377–1382. 14528297 10.1038/nm942

[37] Frank I, Budde C, Fiedler M, Dahmen U, Viazov S, et al (2007) Acute resolving woodchuck hepatitis virus (WHV) infection is associated with a strong cytotoxic T-lymphocyte response to a single WHV core peptide. J Virol 81: 7156–7163. 17459928 10.1128/JVI.02711-06 PMC1933276

[38] Miller DS, Boyle D, Feng F, Reaiche GY, Kotlarski I, et al (2008) Antiviral therapy with entecavir combined with post-exposure “prime-boost” vaccination eliminates duck hepatitis B virus-infected hepatocytes and prevents the development of persistent infection. Virology 373: 329–341. 18206204 10.1016/j.virol.2007.11.032

[39] Inchauspe G, Bach G, Martin P, Bonnefoy JY (2009) Vaccination against hepatitis B and C: towards therapeutic application. Int Rev Immunol 28: 7–19. 19241251 10.1080/08830180802488436

[40] Inchauspe G, Michel ML (2007) Vaccines and immunotherapies against hepatitis B and hepatitis C viruses. J Viral Hepat 14 Suppl 1: 97–103. 17958650 10.1111/j.1365-2893.2007.00922.x

[41] Chisari FV, Ferrari C (1995) Hepatitis B virus immunopathogenesis. Annu Rev Immunol 13: 29–60. 7612225 10.1146/annurev.iy.13.040195.000333

[42] Fourel G, Couturier J, Wei Y, Apiou F, Tiollais P, et al (1994) Evidence for long-range oncogene activation by hepadnavirus insertion. EMBO J 13: 2526–2534. 8013453 10.1002/j.1460-2075.1994.tb06542.x PMC395126

[43] Patient R, Hourioux C, Sizaret PY, Trassard S, Sureau C, et al (2007) Hepatitis B virus subviral envelope particle morphogenesis and intracellular trafficking. J Virol 81: 3842–3851. 17267490 10.1128/JVI.02741-06 PMC1866106

[44] Yuki N, Nagaoka T, Yamashiro M, Mochizuki K, Kaneko A, et al (2003) Long-term histologic and virologic outcomes of acute self-limited hepatitis B. Hepatology 37: 1172–1179. 12717399 10.1053/jhep.2003.50171

[45] Martinot-Peignoux M, Lapalus M, Asselah T, Marcellin P (2013) The role of HBsAg quantification for monitoring natural history and treatment outcome. Liver Int 33 Suppl 1: 125–132. 23286856 10.1111/liv.12075

[46] Vigano M, Lampertico P (2012) Clinical implications of HBsAg quantification in patients with chronic hepatitis B. Saudi J Gastroenterol 18: 81–86. 22421711 10.4103/1319-3767.93805 PMC3326981

[47] Lu M, Hilken G, Kruppenbacher J, Kemper T, Schirmbeck R, et al (1999) Immunization of woodchucks with plasmids expressing woodchuck hepatitis virus (WHV) core antigen and surface antigen suppresses WHV infection. J Virol 73: 281–289. 9847331 10.1128/jvi.73.1.281-289.1999 PMC103832

[48] Schlereth B, Germann PG, ter Meulen V, Niewiesk S (2000) DNA vaccination with both the haemagglutinin and fusion proteins but not the nucleocapsid protein protects against experimental measles virus infection. J Gen Virol 81: 1321–1325. 10769075 10.1099/0022-1317-81-5-1321

[49] Richman LK, Klingenstein RJ, Richman JA, Strober W, Berzofsky JA (1979) The murine Kupffer cell. I. Characterization of the cell serving accessory function in antigen-specific T cell proliferation. J Immunol 123: 2602–2609. 91637

[50] Wiltrout RH, Mathieson BJ, Talmadge JE, Reynolds CW, Zhang SR, et al (1984) Augmentation of organ-associated natural killer activity by biological response modifiers. Isolation and characterization of large granular lymphocytes from the liver. J Exp Med 160: 1431–1449. 6491601 10.1084/jem.160.5.1431 PMC2187504

[51] Zelinskyy G, Dietze K, Sparwasser T, Dittmer U (2009) Regulatory T cells suppress antiviral immune responses and increase viral loads during acute infection with a lymphotropic retrovirus. PLoS Pathog 5: e1000406. 19714239 10.1371/journal.ppat.1000406 PMC2727466

[52] Lu M, Isogawa M, Xu Y, Hilken G (2005) Immunization with the gene expressing woodchuck hepatitis virus nucleocapsid protein fused to cytotoxic-T-lymphocyte-associated antigen 4 leads to enhanced specific immune responses in mice and woodchucks. J Virol 79: 6368–6376. 15858020 10.1128/JVI.79.10.6368-6376.2005 PMC1091665

[53] Bruni R, Conti I, Villano U, Giuseppetti R, Palmieri G, et al (2006) Lack of WHV integration nearby N-myc2 and in the downstream b3n and win loci in a considerable fraction of liver tumors with activated N-myc2 from naturally infected wild woodchucks. Virology 345: 258–269. 16271377 10.1016/j.virol.2005.09.061

[54] Sambrook J FE, Maniatis T, editor(1989) Molecular Cloning. A Laboratory Manual. 2nd ed. Cold Spring Harbor (NY): Cold Spring Harbor Laboratory Press p. 9.31–9.55.

[55] Gerlich WH, Wend U, Glebe D (2004) Quantitative assay of hepatitis B surface antigen in serum or plasma using laurell electrophoresis. Methods Mol Med 95: 57–63. 14762296 10.1385/1-59259-669-X:57

[56] Tolle TK, Glebe D, Linder M, Linder D, Schmitt S, et al (1998) Structure and glycosylation patterns of surface proteins from woodchuck hepatitis virus. J Virol 72: 9978–9985. 9811735 10.1128/jvi.72.12.9978-9985.1998 PMC110511

