# Supplementary figures and images for: Combination of DNA Prime – Adenovirus Boost Immunization with Entecavir Elicits Sustained Control of Chronic Hepatitis B in the Woodchuck Model
Source: PLoS Pathog. 2013 Jun 13;9(6):e1003391. doi: 10.1371/journal.ppat.1003391 (PMC3681757; doi:10.1371/journal.ppat.1003391)

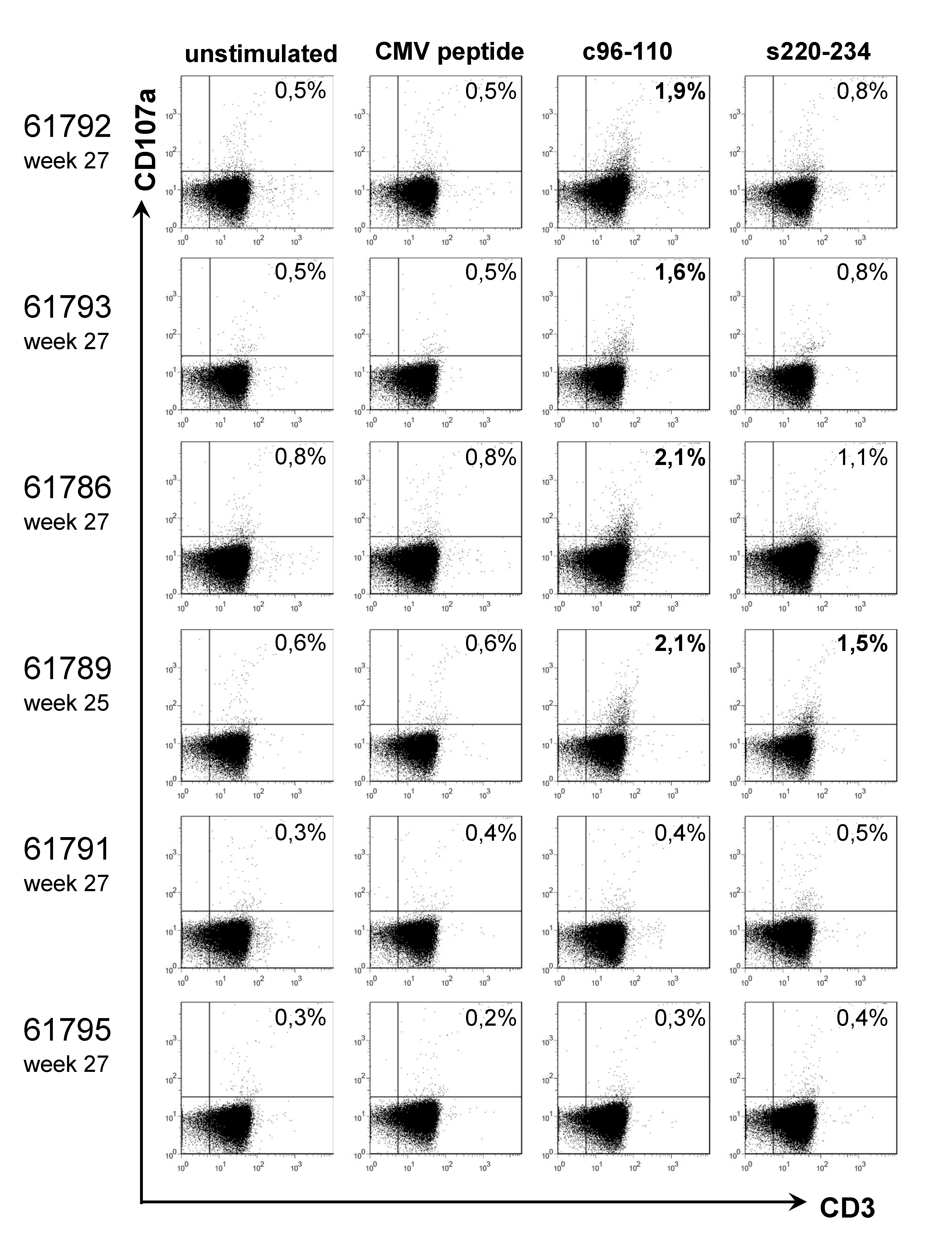

Supplement: Figure S2 — Representative dot-plots of CD107a+ degranulation responses detected in treated WHV chronic carriers. Woodchucks number 61786, 61789, 61792 and 61793 were treated with combination therapy (ETV+ vaccine). Woodchucks 61791 and 61795 were treated only with ETV and served as controls. PBMCs were expanded in vitro for 3 days with WHcAg-derived epitope c96-110 or WHsAg-derived epitope s220-234. Unstimulated cells and cells stimulated with unrelated CMV-derived peptide served as a negative controls. Presented values shows the percentage of CD107a+ CD3+ CD4− T-cells in the CD3+ CD4− T-cell population. (TIFF) [file ppat.1003391.s002.tiff]
