# Supplementary material for: Combination of DNA Prime – Adenovirus Boost Immunization with Entecavir Elicits Sustained Control of Chronic Hepatitis B in the Woodchuck Model
Source: PLoS Pathog. 2013 Jun 13;9(6):e1003391. doi: 10.1371/journal.ppat.1003391 (PMC3681757; doi:10.1371/journal.ppat.1003391)
Supplement: Table S1 — Positive WHsAg- and WHcAg-specific lymphoproliferative responses (Stimulation index ≥3,0) detected in chronically WHV-infected woodchucks during the therapy. (DOC) [file ppat.1003391.s003.doc]

| **Antigen** | **Epitope** | **Week of the therapy** | **Woodchuck number** | **Stimulation index** |
| --- | --- | --- | --- | --- |
| WHsAg | s224-239 | 12 | 61792 | 6,8 |
|  |  |  | 61793 | 17,9 |
|  |  | 19 | 61786 | 3,0 |
|  |  |  | 61789 | 6,1 |
|  |  |  | 61792 | 3,0 |
|  |  | 25 | 61792 | 3,9 |
|  | s252-267 | 12 | 61792 | 3,2 |
|  |  | 14 | 61786 | 3,8 |
|  |  |  | 61789 | 3,6 |
|  |  |  | 61792 | 3,2 |
|  |  |  | 61793 | 3,5 |
|  |  | 16 | 61789 | 5,0 |
|  | s336-351 | 6 | 61793 | 6,8 |
|  | s392-407 | 19 | 61789 | 7,7 |
|  |  | 25 | 61789 | 4,7 |
|  | s430-431 | 12 | 61792 | 5,2 |
|  |  |  | 61793 | 22,4 |
|  |  | 16 | 61792 | 3,6 |
|  |  |  | 61793 | 3,5 |
|  |  | 19 | 61789 | 3,8 |
| WHcAg | c64-79 | 16 | 61789 | 3,0 |
|  |  | 22 | 61786 | 6,7 |
|  |  |  | 61789 | 5,4 |
|  | c78-93 | 14 | 61789 | 3,2 |
|  |  | 16 | 61793 | 3,4 |
|  | c85-100 | 12 | 61792 | 3,0 |
|  | c109-124 | 22 | 61786 | 4,0 |
|  |  |  | 61793 | 3,0 |
|  | c117-132 | 14 | 61793 | 3,4 |
|  |  | 22 | 61786 | 3,2 |
|  |  | 25 | 61786 | 3,7 |
