# Supplementary material for: Combination of DNA Prime – Adenovirus Boost Immunization with Entecavir Elicits Sustained Control of Chronic Hepatitis B in the Woodchuck Model
Source: PLoS Pathog. 2013 Jun 13;9(6):e1003391. doi: 10.1371/journal.ppat.1003391 (PMC3681757; doi:10.1371/journal.ppat.1003391)
Supplement: Table S2 — Amino acid sequence of WHcAg-derived peptides used for in vitro stimulation of woodchuck lymphocytes (Proliferation assay). (DOC) [file ppat.1003391.s004.doc]

| **Position** | **Sequence** |
| --- | --- |
| c1-16 | MDIDPYKEFGSSYQLL |
| c8-23 | EFGSSYQLLNFLPLDF |
| c64-79 | ELTKLIAWMSSNITSE |
| c71-86 | WMSSNITSEQVRTIIV |
| c78-93 | SEQVRTIIVNHVNDTW |
| c85-100 | IVNHVNDTWGLKVRQS |
| c93-108 | WGLKVRQSLWFHLSCL |
| c101-116 | LWFHLSCLTFGQHTVQ |
| c109-124 | TFGQHTVQEFLVSFGV |
| c117-132 | EFLVSFGVWIRTPAPY |
