# Supplementary material for: Combination of DNA Prime – Adenovirus Boost Immunization with Entecavir Elicits Sustained Control of Chronic Hepatitis B in the Woodchuck Model
Source: PLoS Pathog. 2013 Jun 13;9(6):e1003391. doi: 10.1371/journal.ppat.1003391 (PMC3681757; doi:10.1371/journal.ppat.1003391)
Supplement: Table S3 — Amino acid sequence of WHsAg-derived peptides used for in vitro stimulation of woodchuck lymphocytes (Proliferation assay). (DOC) [file ppat.1003391.s005.doc]

| **Position** | **Sequence** |
| --- | --- |
| s210-225 | MSPSSLLGLLAGLQVV |
| s224-239 | VVYFLWTKILTIAQNL |
| s238-253 | NLDWWWTSLSFPGGIP |
| s252-267 | IPECTGQNSQFQTCKH |
| s266-281 | KHLPTSCPPTCNGFRW |
| s280-295 | RWMYLRRFIIYLLVLL |
| s294-309 | LLLCLIFLLVLLDWKG |
| s308-323 | KGLIPVCPLQPTTETT |
| s322-337 | TTVNCRQCTLSVQDTY |
| s336-351 | TYTPPYCCCLKPTAGN |
| s350-365 | GNCTCWPIPSSWALGN |
| s364-379 | GNYLWEWALARFSWLN |
| s378-393 | LNLLVPLLQWLGGISL |
| s292-407 | SLIAWFLLIWMIWFWG |
| s406-421 | WGPALLSILPPFIPIF |
| s420-431 | IFVLFFLIWVYI |
